# Algal-based bioplastics: global trends in applied research, technologies, and commercialization

**DOI:** 10.1007/s11356-024-33644-9

**Published:** 2024-05-24

**Authors:** Trisha Mogany, Virthie Bhola, Faizal Bux

**Affiliations:** Institute for Water and Wastewater Technology, Durban University of Technology, Durban, 4001 South Africa

**Keywords:** Bioploymers, Biodegradable, Biomass, Polyhydroxyalkanoates, Circular economy, SDGs

## Abstract

The excessive global demand for plastic materials has resulted in severe plastic waste pollution. Conventional plastics derived from non-renewable fossil fuels are non-biodegradable, leading to significant environmental problems. Algal-based bioplastics represent a more viable, renewable, and sustainable alternative to conventional plastics. They have identical properties and characteristics as conventional plastics while being naturally biodegradable. The potential of the algal biomass value chain has already been well-established by researchers. Here, we review the novel insights on research, technology, and commercialization trends of algal-based bioplastics, encompassing macroalgae and green microalgae/cyanobacteria. Data showed that within the last decade, there has been substantial interest in utilizing microalgae for biopolymer production, with more focus on using cyanobacterial species compared to green algae. Moreover, most of the research conducted has largely focused on the production of PHA or its co-polymers. Since 2011, there have been a total of 55 patents published related to algal-based bioplastics production. To date, ~ 81 entities worldwide (commercial and private businesses) produce bioplastics from algae. Overall results of this study emphasized that even with the economic and social challenges, algae possess a substantial potential for the sustainable development of bioplastics while also addressing the UN’s SDGs.

**Supplementary Information:**

The online version contains supplementary material available at 10.1007/s11356-024-33644-9.

## Introduction

Over the years, the huge reliance on synthetic plastics (fossil-based materials) owing to the relatively cheap production costs and wide range of applications has led to a dire environmental crisis (Gomes et al. 2020; Kapustin and Grushevenko 2023; Onen Cinar et al. 2020). These conventional plastics persist in the environment for extended periods, contributing significantly to “white pollution” across oceans globally (Dang et al. 2022; López Rocha et al. 2020). The current accumulation of plastic waste is estimated at 4.8 to 12.7 million tons annually, coupled with a projected 11 billion tons by 2025 (Roja et al. 2019; Brahney et al. 2020). Plastic pollution adversely affects marine life and ecosystems, leading to entanglement, suffocation, and starvation of hundreds of thousands of marine animals. Moreover, macro-, meso-, micro-, and nano-plastics that enter the food chain pose a direct threat to human health, with seafood consumption introducing substantial amounts of microplastics containing toxic chemicals (Chia et al. 2020; Cheah et al. 2023).

Increasing oil prices and supply of conventional plastics, as well as restrictions on landfilling, the limitations of current waste management practices, and the escalating trajectory of plastic production have motivated the research and development (R&D) of plastic alternatives (Di Bartolo et al. 2021). Within the last decade, several governments, industries, and policymakers have initiated measures, including legal restrictions and taxes on single-use plastics, to limit the environmental impact of conventional plastics and transition towards more eco-friendly alternatives (Rosenboom et al. 2022; Schmidtchen et al. 2022; Ghaffar et al. 2022; United Nations Environment Program  2018). Several countries have also committed to the development of natural plastic alternatives to support United Nations Sustainable Development Goals (UN SDGs) by reducing the dependence on conventional plastics and promoting a shift towards green alternatives contributing to climate change mitigation (Moshood et al. 2021).

Natural plastic alternatives known as bioplastics are biodegradable, biobased materials derived from renewable resources, and are made through biological processes or a combination of these (Abrha et al. 2022; Ibrahim et al. 2021; Liu 2022a). The European Union (EU) has recognized bioplastics as a promising alternative to conventional plastics. However, it is essential to find cost-efficient biobased materials with reduced environmental footprints that fit into the circular economy (Rosenboom et al. 2022). Among the alternatives, algal-based bioplastics have emerged as a promising solution. Third-generation bioplastics derived from algal biomass and biomaterials from bacterial polymers from industrial or municipal waste offer innovative feedstocks that are still at an early stage of development (Coppola et al. 2021). Algae compromising both microalgae and macroalgae potentially offer a sustainable and high production of biomass. Unlike bioplastics produced from terrestrial plants that contribute to deforestation and compete with essential agricultural lands, algae can be cultivated on non-arable lands, minimizing the impact on food and feed industries (Mathiot et al. 2019; Price et al. 2020). Algae not only utilize CO_2_ as a nutrient for biomass production but also have the potential to capture and permanently store CO_2_ in the biomass (Chia et al. 2020; Ummalyma et al. 2021a). Hence, the reduced environmental footprint of algal bioplastics is marked by their capacity to absorb CO_2_ during growth, contributing to a net reduction in greenhouse gas emissions. Importantly, these bioplastic materials derived from algae decompose much more easily and faster mitigating the persistent negative environmental impact associated with traditional plastics.

Furthermore, most algal strains adapt to fluctuating environment conditions. When exposed to stress conditions, algae can easily undergo significant physiological reorientations leading to the accumulation of intracellular products such as lipids, cellulose, starch, and polymers (Mathiot et al. 2019). In contrast, plant-based feedstocks have layered cell walls making it difficult to extract compounds, especially polymers for the synthesis of bioplastics. Furthermore, bioplastics manufactured from food crops such as corn, sago, and cassava have demonstrated poor mechanical properties and water resistance (Chia et al. 2020). Algal biomass characterized by long-chain hydrocarbons and a low percentage of lignin makes it a favorable feedstock for bioplastics production (Zanchetta et al. 2021). Algae are gaining importance in the framework of bioeconomy as a source of biobased plastics, and more research is focused on the application of algae as a new sustainable way to alternative plastics for our daily activities.

Like any other novel approach, there are certain limitations associated with producing algae-based bioplastics. However, there have been considerable amounts of research and pilot-scale projects conducted on this topic, and several commercial companies are currently successfully producing these bioplastics, which highlights that algal-based bioplastic production is a promising alternative. Breakthroughs in algae-based bioplastics include advancements in genetic modification for increased biopolymer yields (Kamravamanesh et al 2018; Kim et al 2020; Katayama et al. 2018; Roh et al 2021), engineering for enhanced mechanical properties (Semary et al. 2022; Zhu et al. 2017), and novel applications in 3D printing (Mandal et al. 2023; Vo et al 2022), highlighting the dynamic progress and multidisciplinary nature of research in this field.

This review, therefore, aims to provide novel insights into algae-based bioplastics by analyzing the technological trends using publications, research projects, and patent information in the last ± 10 years. This review seeks to identify and analyze emerging technologies that have the potential to shape the future of algal-based bioplastics. An integral objective of this review is to emphasize the opportunities for research and development (R&D) in the field of algal-based bioplastics. By examining recent advancements and breakthroughs, the review aims to highlight the aspects where innovation can contribute to the development of more sustainable and advanced algal-based bioplastic.

Within the scope of this study, we performed a SWOT analysis to explore challenges and examined the potential role of algae bioplastics in meeting the UN SDGs. A specific focus is placed on profiling different companies worldwide that are actively engaged in the manufacturing of bioplastics derived from both microalgal and macroalgal biomass. This provides a practical dimension to the review by highlighting real-world applications and initiatives and offering insights into the current state of the industry and potential collaboration opportunities.

## Methodology

### SWOT analysis

Within the scope of the study, a SWOT analysis was performed to explore the strengths, weaknesses, opportunities, and threats associated with algal-based bioplastics. This strategic analysis provides a comprehensive understanding of the internal and external factors influencing the development and adoption of algal bioplastics. The results of this study were based on the available information collected from related publications and websites cited throughout this paper. A modified version of the SWOT analysis framework (Ommani 2011) was thereafter employed to analyze the information and identify areas of strengths and weaknesses of algae as a feedstock for bioplastic production. By using the SDGs as a unifying platform, we were also able to forecast areas of opportunities for algae-based bioplastics as well as recognize threats/uncertainties that could pose problems for the development of this technology.

### Literature search

The protocol applied was a systematic web search for any information that reported on companies utilizing algal biomass to develop bioplastic components/products. This report aimed to outline the different types of evidence on the particular area of interest and gaps that necessitate further research. The search strategy was conducted in three phases: database search, filtering, and analysis. The following five steps were therefore followed to the best of our ability: (i) identifying the research question; (ii) identifying the research conducted, applicable companies, and technologies in the last decade (± 10 years); (iii) selection of eligible companies and patents; (iv) reporting on the findings; and (v) collating and summarizing the findings.

#### Research studies

The Web of Science, Scopus, Google Scholar, and Science Direct databases were selected for analyzing data on algal bioplastic research. Scientific articles published between January 2012 and December 2022 were included in the search. Different combinations of the keywords—namely, “algae bioplastics,” “cyanobacteria bioplastics,” “algae Polyhydroxyalkanoates (PHAs),” “cyanobacteria PHAs,” “algae Polyhydroxybutyrates (PHBs),” “cyanobacteria PHBs,” “biopolymers,” “algae-based materials,” and “cyanobacteria-based materials”—were employed for the electronic search strategy. The potential articles from each database were identified; duplicates were removed, and relevant articles were then scanned to confirm relevance. Following the exclusion of duplicates, records returned from this search were then screened using a set of eligibility criteria.

Eligibility criteria for selected articles were as follows: (i) studies published as an original article with full text and (ii) studies that specifically investigated the potential of microalgae/macroalgae for bioplastic/biopolymer production. The exclusion criteria of the search were as follows: (i) manuscripts that were secondary research, such as review articles, systematic reviews, or conference proceedings; (ii) articles that were not available in English; (iii) articles that did not provide sufficient information; (iv) manuscripts that were older than 2010; (v) the topic of the article was not related to the keywords; (vi) algal biopolymers for clinical or food applications. Any “grey” information was also excluded from the search as there were concerns regarding the peer-review process and the possibility that some “grey” information would not provide sufficient information that could be included in the final review. Furthermore, to identify additional manuscripts of interest that may have been missed during the electronic search, the reference lists from the publications obtained were also screened. EndNote X9 (Clarivate, Philadelphia) was used to store, to remove duplicates, and screen references. Excel (Microsoft Office 365) was utilized for the screening and data extraction stages of the review process.

#### Companies and technologies: database patent search and data analysis

To start our analysis of algae bioplastic research projects, companies, and patents, the search was applied to three different databases, such as Espacenet (https://worldwide.espacenet.com/), Lens (https://www.lens.org/), and Google patents (https://www.google.com/patents) from January 2010 to December 2022. Patents were selected based on patent abstracts related to algae-based bioplastics. The documents were examined in full and analyzed according to the patent title, office, publication number, publication date, grant number, grant date, patent status, country, applicants, and inventors (Supplementary data Table S1). The redundant patents that appeared in more than one database were excluded from the analysis to avoid result misinterpretation. A filtering step was employed since the same patent may be found in more than one database and can be registered in several offices.

## SWOT analysis: algal-based bioplastics

**Fig. 1:**
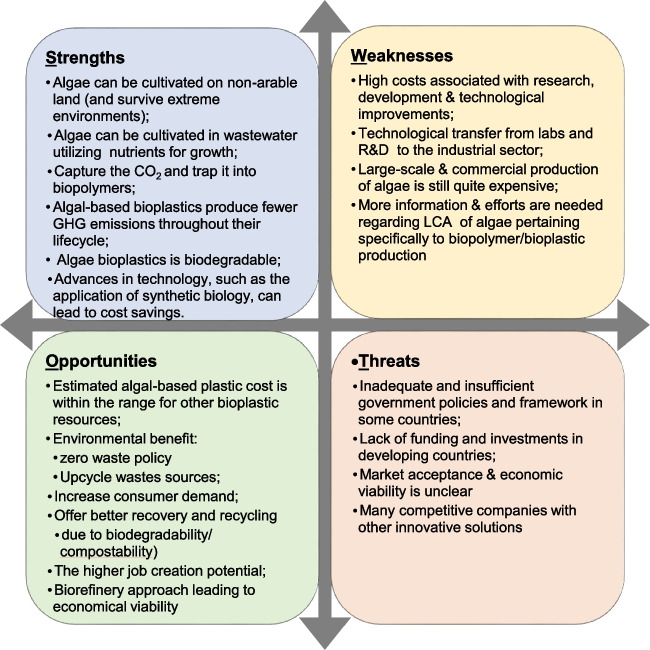
SWOT analysis for algal-based bioplastic production

**Fig. 2:**
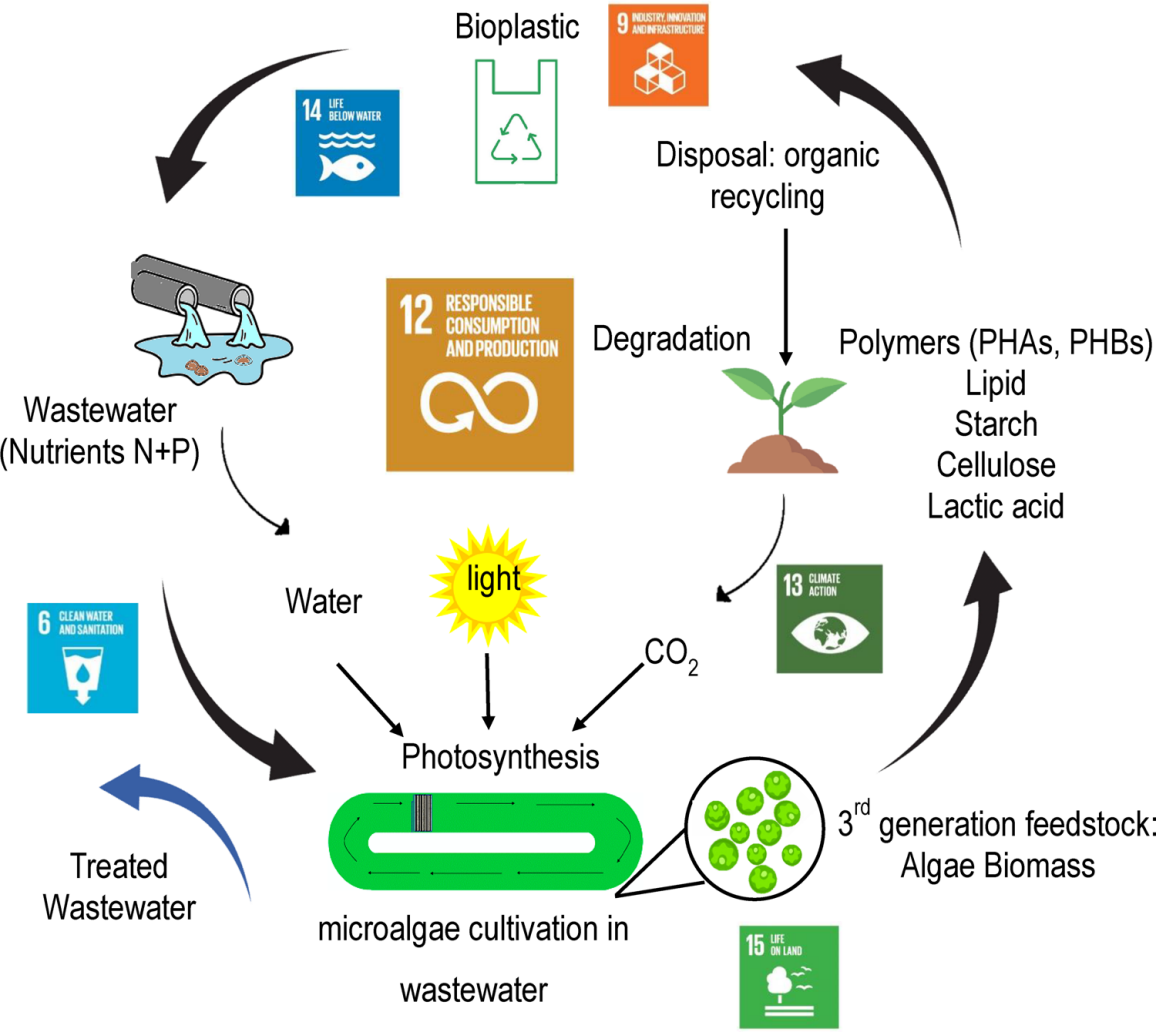
Algal biobased plastic leading to a circular economy and sustainable future

The SWOT analysis shows that there are substantial amounts of strengths and opportunities for algae to be potential as feedstock for the production of bioplastics; however, there are still considerable weaknesses and possible threats that are limiting the development of the algal bioplastic industry (Fig. 1). The SWOT analysis provides an appraisal of the current perspective and outlook of the global algal-based bioplastic market. We also examine and discuss the potential role of algae bioplastics in meeting the UN SDGs, specifically considering SDG 12 which focuses on decreasing the production and consumption of conventional plastics as well as SDG 6 to improve water quality, SDG 13 to mitigate climate change, SDG 14 to protect the ocean, and SGD 15 to protect the land (Fig. 2).

### Strengths of algae as a resource and algae-based bioplastics

#### Use of non-arable land

Sustainable algal cultivation not only presents an environmentally friendly alternative but also addresses concerns related to land use. Microalgae and macroalgae can be cultivated on non-arable land, which eliminates the competition with agricultural food production and deforestation. Macroalgae are grown in marine environments primarily near shore and have minimal resource requirements (Sudhakar et al. 2018). Karan et al. (2019) reported that utilizing algae for global bioplastic production would require approximately 0.028% of the Earth’s surface area, highlighting the potential for efficient land use.

#### Efficient resource utilization

Moreover, regarding the efficiency of raw material to product conversion, algae often have ± 90% conversion efficiency with an almost equal mass conversion to bioplastic (Karan et al. 2019)*.* Microalgae are versatile photosynthetic microorganisms that can be grown in various environments including saline and wastewater. They efficiently utilize the inorganic and organic nutrients (carbon, nitrogen, and phosphorus) from the wastewater and assimilate them as part of their biomass (Ummalyma et al. 2021a). Thus, microalgae can potentially be used for recovering resources from the waste resulting in cleaner water for beneficial reuse in an economic way, highlighting SDG-6 “Clean Water and Sanitation” (Bhatt et al. 2022; Oliveira et al. 2022). Successful large-scale demonstrations, reported by Acién Fernández et al. (2018), Aljabri et al. (2020) and Arbib et al. (2022), highlight the practical application of microalgae in resource recovery from wastewater. Furthermore, cultivation medium costs could be significantly reduced if wastewater resources were used instead of chemical-based components (Catone et al. 2021; Devadas et al. 2021).

#### Lower greenhouse gas emissions

Algae-based bioplastics offer a significant advantage in terms of reducing the carbon footprint associated with plastic production. Algae-based bioplastics leverage photosynthesis to convert carbon dioxide into biomass; this process actively captures and sequesters carbon during the cultivation of algae. Algae possess strong CO_2_ assimilation abilities and can grow at high concentrations of CO_2_ (40%) (Akash et al. 2023). Typically, 1 kg of algae can assimilate roughly 1.83 kg of atmospheric CO_2_ with a fixation rate ranging from 0.73 to 2.22 g/L/day (Molazadeh et al. 2019). Microalgae have a carbon fixation capacity 10 to more than 50 times higher than other plants (Ighalo et al. 2022; Prasad et al. 2021; Yadav et al. 2020).

Consequently, the carbon footprint of algae-based bioplastics is substantially lower compared to traditional plastics. Moreover, algae-based bioplastic has proven significant in producing fewer greenhouse gas emissions compared to traditional synthetic plastics (Roy Chong et al. 2022). This contributes to SDG 13 which focuses on the awareness of climate change and global warming. The environmental impact of polylactic acid (PLA) from microalgae contributes less to eco-toxicity compared to plant-based PLA as reported by the LCA assessment by Bussa et al. (2019).

#### Diverse metabolite composition

Microalgae and macroalgae both offer unique strengths in metabolite composition contributing distinct advantages for the production of bioplastics. Microalgae can produce a variety of metabolites, including proteins, carbohydrates, and lipids, offering versatility in the types of bioplastics that can be derived (Roy et al. 2022). Macroalgae typically comprise 50 to 76% of their dried weight in polysaccharides, featuring natural derivatives like starch, agar, alginate, cellulose, carrageenan, fucoidan, and ulvan, which serve as natural binding agents and demonstrate film-forming properties (Farghali et al. 2023).

#### Algae-based bioplastic properties and applications

Bioplastics derived from algal biomass can be manufactured using three techniques: (i) the direct utilization of algal biomass, (ii) through the extraction of polymers and other compounds (iii) by blending algal biomass with other materials such as additives and polymers. Genetic engineering tools have also been applied to manipulate and create ideal polymer-producing algal strains (Chia et al. 2020).

Zeller et al. (2013) reported the direct production of thermoplastic blends and bioplastics from *S. platensis* and *C. vulgaris* and found that blending is required for commercial applications. Similarly, Wang et al. (2016) demonstrated the synthesis of thermoplastics by mixing a variety of planktonic algae. Although direct methods exist, the most common technique involves incorporating microalgal biomass with conventional plastics such as polyethylene, polypropylene, and polyvinyl chloride.

Shi et al. (2011) used *Nannochloropsis* and *Spirulina* to manufacture microalgae-corn starch-based thermoplastics in an inventive way, combining them with polyethylene and polypropylene. Zhang et al. (2000) combined *Chlorella* sp. biomass with polyethylene and polypropylene and revealed good thermoplastic processability owing to the cellulose components present. The addition of a 6 wt% compatibilizer into *S. platensis* and polyvinyl alcohol (PVA) mixture resulted in a bioplastic film with higher tensile strength compared to commercial plastic bags, as well as enhanced elongation capability and smoother surfaces, whereas the addition of 30% glycerol into *S. platensis* and PVA produced bioplastic and can be used for food packaging, in pharmaceutical applications. The blending of macroalgae with additives such as plasticizers is still required to enhance the mechanical properties of the bioplastics. Microalgae biomass can be effectively blended with PLA, a commercially produced bioplastic derived entirely from biological sources (Carina et al., 2021). Polylactic acid exhibits properties that are similar and have comparable tensile strengths and melting temperatures to polystyrene, polyethylene, and polypropylene. PHB is relatively brittle compared to PLA, which has better flexibility, however, still lower than traditional polyolefins. The addition of plasticizers can improve the flexibility of both polymers, addressing any brittleness concerns (Kartik et al. 2021; Kato 2019; Simonazzi et al. 2021). More importantly, PLA, PHA, starch, and protein could also be obtained from microalgae.

A recent meta-review by Yap et al. (2023) reported that most algal-based bioplastic films have good mechanical properties with regard to tensile strength (MPa), elongation at break (%), and water vapor permeability (WVP × 1010) (g/m/s/Pa) in edible, non-edible, and biodegradable packaging applications. However, algal-based bioplastic films have a wide range of permeability to water contrasting to commercial plastics characterized by lower WVP which act as good barrier packaging material.

Algae-based bioplastics can be engineered to possess specific properties, making them suitable for a wide range of applications, including packaging, textiles, the biomedical industry, and even 3D printing (Kartik et al. 2021). These approaches highlight the diverse strategies available for using microalgae in the production of environmentally friendly bioplastics.

### Opportunities integrated with SDGs

#### Waste reduction and sustainable end-of-life

Algal-based bioplastics contribute to SDG 12 by offering a sustainable end-of-life cycle. Algal-based bioplastics decrease the amount of plastic pollutants from the manufacturing process to consumption and inappropriate disposal, as well as reduce the burden on landfills. Unlike conventional plastics, which cannot naturally break down and remain in the environment for a long time, algal-based bioplastics produced from PHAs and other polysaccharides naturally degrade into CO_2_, water, and biomass, supporting a circular economy approach (Chia et al. 2020; Costa et al. 2019; Karan et al. 2019). Bioplastics made from biopolymers such as PLA, PVA, and starch can be biodegraded by soil microorganisms within 30–180 days under industrial composting conditions (Kalita et al. 2021; Pantelic et al. 2021). As these materials break down, they leave behind minimal ecological impact, reducing the accumulation of plastic waste and its associated environmental consequences. They could also be applied as a peat substitution which would terminate the life cycle of the bioplastic (Devadas et al. 2021). The rapid degradation of macroalgae-based bioplastics within 4 weeks, as reported by Lim et al. (2021), highlights their potential for swift and eco-friendly disposal. It has been suggested that the algae-based end products could potentially be used as agricultural films or as fertilizer to help maintain soil moisture (Schmidtchen et al. 2022). The biodegradable nature of this aliphatic polyester enables a closed-loop cycle from cradle to cradle, thus minimizing the impact on the environment (Meereboer et al.^2020^). Considering the low biodegradability of conventional plastic, algal-based bioplastics provide a significant advantage with regard to environmental benefits.

#### Marine pollution mitigation

By avoiding the release of toxic contaminants such as microplastics into the environment, algae-based bioplastics directly contribute to SDG 14, minimizing marine pollution and protecting oceans and marine biodiversity (Sutherland et al. 2021). The biodegradability of algae-based bioplastics further reduces the impact of plastic waste on oceans and seas (Ghaffar et al. 2022).

#### Biodiversity conservation and resource management

Algae-based bioplastics contribute to SDG 15 by mitigating eutrophication, reducing ocean acidification, capturing/sequestering CO_2_, and avoiding risks of biodiversity reduction or potential deforestation. Research by Schmidtchen et al. (2022) and Oliveira et al. (2022) support the idea that the production of algae-based bioplastics does not pose a risk to biodiversity, aligning with the sustainable management of natural resources.

#### Economic viability and cost competitiveness

Although the current total production volumes of bioplastics are only 1% of annual plastics manufacturing, the market potential is expected to increase. The current market data research for European Bioplastic suggests that bioplastic production is estimated to increase from ~ 2.11 million tons in 2020 to 2.87 million tons in 2025 (Mal et al. 2022; Mehta et al. 2021). According to the “The Global Bioplastics Market Outlook 2018–2028” report, in 2021, bioplastics had a global market value of ~ 7.6 billion USD, and it is expected to reach 15.55 billion USD by 2028.

The versatility of algae-based bioplastics allows for the replacement of various traditional plastics at competitive production costs. Estimated annual production costs for algal-based plastic are USD 970/ton, which is within the projected range for other bioplastic resources (Beckstrom et al. 2020). These include commodity thermoplastics (USD 1540–2200/ton), biodegradable resins (USD 2640–5500/ton), and engineered resins (USD 1540–8800/ ton). Although the current, large-scale algal cultivation methods are resource intensive, future economic and ecological pressures, coupled with environmental benefits, make these tradeoffs worthwhile. Addressing the use of costly raw materials and scaling up production processes are key areas where advancements are required. A reduction in the final cost of bioplastic packaging is expected to drive significant growth within the bioplastic market. Consumer willingness to adopt green products and a positive attitude towards sustainability contribute to the positive outlook for the economic feasibility of algal-based bioplastics (Nanda and Bharadvaja 2022). This supports the potential for market competitiveness and sustainable choice in the broader landscape of bioplastic alternatives.

### Weakness and threats

#### Cost

The main issue with economic and market potential is that bioplastics have been reported to be 2 to 5 times more expensive than conventional plastics (Folino et al. 2020). This is mainly due to high R&D cost, high cost of raw materials, and small production scale. Although there has been a positive attitude of customers towards sustainability and an increase in them willing to purchase environmentally friendly products, there is limited evidence to suggest that the purchase of these products has increased (Dilkes-Hoffman et al. 2019). Reducing the manufacturing cost will decrease the final cost of bioplastic packaging, driving the bioplastic market growth.

#### Durability and barrier function

Natural macroalgae polymer is not be water resistant, thus providing lower durability and barrier function compared to conventional plastics (Lomartire et al. 2022). However, such properties are not required for all plastic applications such as non-food packaging or single-use products. In order to make macroalgae-based packaging materials water-resistant, it can be modified by including additives or a coating to improve the material properties (Schmidtchen et al. 2022).

#### Limited biodegradability and mechanical properties

Bioplastics made from PLA and PVA may not biodegrade as effectively in natural environments such as soil or marine water. PLA is capable of degrading efficiently in industrial composting facilities with controlled conditions such as high temperatures and high humidity levels and requires PLA-degrading microorganisms to break down the materials to polymers (Kalita et al. 2021; Pantelic et al. 2021). Thus, direct landfilling of these bioplastics is not feasible. The disposal of these bioplastics via industrial composting is recommended for maximizing the environmental benefits of PLA from algae, which will potentially require land space, a controlled environment, and regular monitoring (Nanda et al 2022; Plavec et al. 2020).

Starch/carbohydrate-based bioplastics are only suitable for single-use items due to limited mechanical properties, poor moisture and thermal stability, high gas permeability, and challenges in processing. However, starch is a good filler to be used in blends (Fabunmi et al. 2007). To improve the properties of starch-based bioplastics, research is being conducted to use nanotechnology-based reinforced materials and ultraviolet irradiations (Nanda et al 2022).

#### Lack of global policies and standards

Currently, only a few countries have policies specifically targeting the bioplastics sector, whereas several countries have policies related to the bioenergy sector, which places bioplastics at a disadvantage in the competition for algal biomass. There are no standard policies and specifications regarding the quality of bioplastics, despite having several certification systems for the compostability of bioplastics (Organization for Economic Co-operation and Development 2013). Thus, it is imperative that standard guidelines, policies, legislation, and certifications are developed and recognized globally (Chia et al. 2020).

#### Food versus plastic debate

Like other sustainable materials, algae have a wide range of applications. Using algae as a resource for bioplastics is the topic of the current “food-versus-plastic” and “fuel-versus-plastics” discussions, which criticize the conversion of potential food or bioenergy resources to bioplastic. Considering the increasing number of people starving or suffering from lack of nutrition, it is debated especially on a larger scale to convert algae with nutritional value to bioplastics. Despite the substantial evidence for algae as nutritional and functional foods, currently, a limited number of algal species, i.e., *Spirulina* sp., *Chlorella* sp., *C. reinhardtii*, *Haematococcus* sp., and *Dunaliella* sp. are internationally recognized as “generally regarded as safe” (GRAS) (Fabris et al. 2020). Algae are not recognized as a food and to date, little or no guidance for approving novel food. Moreover, algae cultivated on waste streams are not considered acceptable for human/animal consumption due to the uptake of toxic pollutants and emerging contaminants (EC) such as pharmaceuticals, herbicides, and pesticides.

Despite receiving the most attention, algal biofuels are still not economically feasible. However, in cases where algae are being cultivated for biofuels, with high lipid content, the lipid can be extracted for biofuels, and residual biomass (composed of proteins, carbohydrates such as cellulose, and polysaccharides) can be used for bioplastic production (Ummalyma et al. 2021b). Algae residual biomass has to be plasticized with polyether and urea, blended with other materials, or chemically modified for further usage in packaging applications (Fabris et al. 2020). The use of algae for the production of biofuels requires a biorefinery approach to enhance the economic feasibility of the fuels. Coupling fuel production with bioplastics could further reduce the use of fossil fuels which are currently used for the manufacture of conventional plastics. Furthermore, utilizing algae for the co-production of biofuel and bioplastics can present a highly promising avenue, particularly when cultivated on waste materials that are deemed unsuitable for consumption, animal feed, or fertilizer applications. .

## An overview of bioplastic research conducted in recent years using algae

**Fig. 3:**
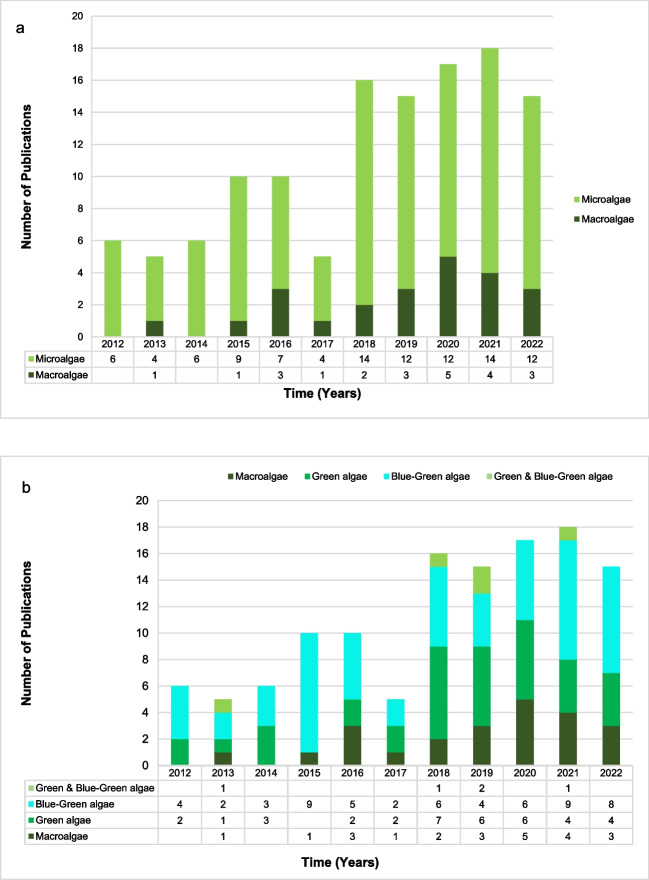
**a** Number of research articles published in the last decade pertaining to the utilization of microalgae and macroalgae for bioplastic production and **b** further subdivision of research articles based on the type of algae exploited

The number of research articles published in each year was extracted. A total of 91 research papers were published between the years 2012–2022 that investigated the use of microalgae/macroalgae for bioplastic/biopolymer production (Fig. 3). For this review, the term microalgae includes microscopic green microalgae and blue-green microalgae, also known as cyanobacteria. Macroalgae which are a polyphyletic group of multicellular algae can also be referred to as seaweeds (Lomartire et al. 2022)*.* Within the past 10 years, there has been considerably more research (99 publications) conducted on the use of microalgal organisms for bioplastic/bio-polymer production as opposed to the utilization of macroalgae/seaweeds (only 22 publications). Macroalgae have the potential to be a source of bioplastics owing to their rapid reproduction leading to higher biomass, and they can also be easily and naturally maintained in all aquatic environments without the need for external nutrients making them cost-effective (Lomartire et al. 2022). However, cultivating them within a control system in laboratories for research purposes is problematic and requires large amounts of space as opposed to microalgae. This could be a possible reason that laboratory and academic-based research over the past decade has focused on employing microalgae over macroalgae for bioplastic/biopolymer production studies.

The number of papers published in the most recent 5 years between 2018 and 2022 surpassed the cumulative publications from all previous years (Fig. 3a). Suggesting algae bioplastics is an emerging field in algal research and gained considerable interest since 2018. This suggests that research initiatives pertaining to bioplastic production from microalgae and macroalgae have escalated within the last 5 years. Similar findings were reported by Madadi et al. (2021) whereby an increase in scientific publications from 2015 related to biomaterials and PHA. The results of the electronic database analysis correlate to an expected increase in global biobased plastic production (Onen Cinar et al. 202). This highlights that the future focus of algal research will shift from basic lab studies to more large-scale applications. The current market data research for European Bioplastic, reported  that bioplastic production is estimated to increase from approximately 2.11 million tons in 2020 to 2.87 million tons in 2025, highlighting the huge demand for biodegradable, polymeric materials (Mal et al. 2022). Increased publication in the area of biodegradable plastics signifies that researchers have identified this as an area that requires development in the short term to meet the industry and legislative requirements. This shift in emphasis highlights the growing potential of microalgae and macroalgae as valuable resources for bioplastic production and a promising trajectory for future advancements in bioplastics R&D.

Furthermore, it is interesting to note the research trend when research activity regarding microalgae (a total of 99 research publications) over the past 10-year period was subdivided into those studies that assessed green algae and those that utilized cyanobacterial species (blue-green algae). From Fig. 3b, it can be seen that most work conducted related to the study of blue-green algae (cyanobacteria) (58 research publications), as opposed to green algae (36 research publications), whilst 5 published studies focused on evaluating both green algae and blue-green algae. The substantial research interest in cyanobacteria in comparison to green algae could be because blue-green algal species are thought to have a slight edge over green algae regarding CO_2_-concentrating mechanisms (CCM) (Raven et al. 2017). For algal species, biopolymer biosynthesis and photosynthesis are closely linked to each other. For efficient photosynthesis to occur, the organism would require a higher expression of CCM activity to effectively fix carbon ultimately leading to higher polymer production (Ciebiada et al. 2020). Based on the increasing number of scientific papers on algae-based bioplastics in the last decade, it can be predicted that a potential increase in the number of new industrial patents on bioplastic production using algae is expected for this time period.

**Fig. 4:**
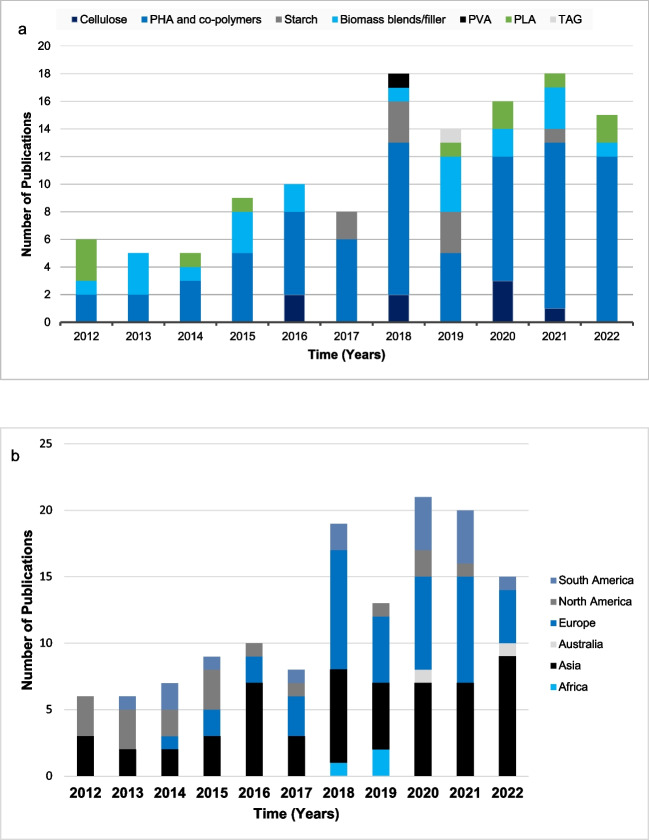
**a** Laboratory scale studies related to products from algae used for bioplastic production from 2012 to 2022 and **b** worldwide distribution of publications since 2012

Research studies dealing with the production of bioplastic material from algal sources can be further categorized into two broad groups. One approach that relies on the intracellular accumulation of biopolymers and starch within algal cells has clearly been the more popular route taken within this stream of research (Fig. 4). The accumulated products can be optimized, thereafter extracted, and processed further for bioplastic production. Cellulose, starch, PHA, PHB, PLA, PVC, and triacylglycerol are examples of well-known compounds produced by algae that can be used for the generation of biodegradable plastics (Chia et al. 2020; Karan et al. 2019). In comparison to other biopolymers, PHAs, and co-polymers such as PHB, poly(3-hydroxybutyrate-co-3-hydroxyvalerate (PHBV), (R)-3-hydroxybutyrate [(R)-3HB], and poly(3-hydroxybutyrate-co-3-hydroxyvalerate) [P(3HB-co-3HV)] are more promising as their material properties are very similar to that of polypropylene, and they can also be processed in a similar manner (injection molding and extrusion) (Costa et al. 2019; Liu et al. 2022a). This is evident from Fig. 4a, which shows that the majority of research conducted within the past 10 years has focused on the production of PHA or its co-polymers, leading to 73 published research articles. In comparison to other compounds, PHA/PHA co-polymers have been the major focus of research for bioplastic applications each year within the past decade. The biodegradable nature of this aliphatic polyester enables a closed-loop cycle from cradle-to-cradle, thus minimizing impact on the environment.

The other approach entails the blending of algal biomass with bio or petroleum-based polymers and additives to produce bioplastic composites. This strategy utilizes the algal biomass as a “filler” and requires methods such as thermal–mechanical (compression molding) techniques to produce the bioplastic product (Cinar et al. 2020). Figure 4a shows that in the past decade, there have been 20 published studies that have investigated this approach to generate bioplastic material. Cost-competitive bioplastics can reach marketability relatively early if the different biomaterials are combined. Thermoplastic starches (TPS) and blends account for the largest market share (60%) among biodegradable bioplastics, owing to their biodegradability, non-toxicity, high purity, low costs, and large availability. Thereafter, PLA has ~ 20% market share, followed by cellulose acetate (CA) with 15% market share (Folino et al. 2020). Other bioplastics such as PHAs are at a market share below 5%, at present. PHA and PLA closely match the characteristics of existing plastics and are at the same time 100% biodegradable. There have only been 8 reported studies within the decade related to starch production from algal sources for bioplastic generation. Similarly, since 2016, there has been limited research interest (8 known publications) in the potential application of algal cellulose for bioplastics.

The geographical distribution of algal bioplastic publications from 2012 to 2022 shows that Asia and Europe are primary regions with 55 and 41 papers published, respectively. The countries with the highest publications are India, the USA, South Korea, Thailand, Italy, and Spain. This global distribution highlights the widespread interest and collaborative efforts in investigating algal-based bioplastics. Asia, particularly India and South Korea, emerges as a pivot center for research, emphasizing the region’s commitment to sustainable solutions. Likewise, European countries, such as Italy and Spain, also significantly contribute to the body of knowledge in the field of algal-based bioplastics. This geographical distribution highlights the worldwide focus of algal-based bioplastic research and the importance of developing a collaborative approach.

## Technologies and innovation

Worldwide, several interventions have attempted to bridge the gap between scientists and the commercial and business aspects. Although universities and research institutes tend to publish their research in literature, patent analysis is also highly important to measure the R&D output and technology evaluation. The integration of intellectual property (IP) offices into universities and commercialization offices is crucial for facilitating the technology evaluation of algae-based bioplastics (Sabri et al. 2018). Some important steps have been taken such as integrating IP offices into universities and building commercialization offices. Universities have implemented technology transfer programs, encouraging collaborations with industry through R&D initiatives (Katzman and Azziz 2021). Innovation hubs and incubators provide environments for startups working on algae-based bioplastics, offering resources and mentorship (Adegbiji 2022). Creating a culture of innovation, IP protection education, and funding opportunities for technology transfer and commercialization is further supported by the government.

This collaboration allows effective management of patents and other IP assets, promoting innovation, protecting discoveries, and enabling a transition from academic research to practical applications in the field of algae-based bioplastics. Furthermore, algal-based bioplastics have not only received discussion in academia but also gained publicity through local startup programs and research projects worldwide to enhance R&D through collaboration between developers and scientists across countries.

Patent protection on new algae-based bioplastic innovations is crucial to attracting investment for further R&D (Preiss and Kowalski 2010). To ensure the protection of industrial innovation, potential inventions are often patented. Therefore, analyzing patents is a valuable approach to identifying developments in a particular field, mainly from the market point of view. Additionally, it provides useful information on the trends, identifying emerging technologies, and planning strategies for research development (Murata et al. 2021). Patents serve as a valuable repository of innovative solutions and emerging technologies, offering insights into the evolving landscape of algal bioplastics. The search of patent databases and algae-based bioplastic innovations during the last 10 years was carried out where the most relevant results and information are presented in Tables 1, 2, and 3. To date, a majority (~ 60%) of the patents published are produced using microalgae as a feedstock, 30% used solely macroalgae, and 10% used both types of biomasses.

**Table 1:** Patents related to bioplastic production using macroalgae

Country	Name of patent	Patent	Inventor/s
China	Edible fully biodegraded seaweed plastic wrap and preparation method thereof	CN104231297A	Xu Jiachao, Hu Yiming, Liang Yunbo, Guo Liling
China	Kelp powder biodegradable plastic and preparation method thereof	CN104829873A	Hefei Ring, Macromolecular Material Factory
China	Method for preparing plastic from algae	CN105331034A	Zhou Fuhai Pan Qi
China	The biological plastics composition for preparing the method for the algae powder of protein content reduction and being prepared with the powder	CN108350178A	
England	A packaging item	WO2021171016A1	Pierre-Yves Paslier, Rodrigo Garcia Gonzalez
France	A biodegradable plastic-like material obtained from a seaweed	WO2016113716A1	Sambhu Bhadra, Mohamed Gouse, Raghavendra, Barki Babu Padmanabhan
Germany	Material based on macroalgae	DE102020103185A1	Ramona Bosse, Frederike Reimold, Laurie Carol Hofmann, Bela H. Buck, Joachim Henjes, Dieter W. HoffmannIna Enders
South Korea	Seaweed fiber-reinforced biocomposite and method for producing the same using high-temperature grinding	EP2079794A1	Seong-Ok HanHong-Soo KimYoon-Jong YooYeong-Bum SeoMin-Woo Lee
South Korea	Red algae-polylactic acid and manufacturing method thereof	KR101237880B1	Seok-gu Seo Young-beom
South Korea	Seaweed-based food packaging coating	KR102233086B1	Steven A. Santos
South Korea	Filament composition for 3-dimensional print comprising red algae fiber	KR20170009425A	Seo Young-beom, Heo Yoon-young
South Korea	Method of manufacturing bio plastic and bio plastic manufactured	KR102270614B1	
South Korea	Eco-friendly plastic bag using seaweed and vegetable raw material and manufacturing method therefor	WO2021101094A1	Cha Wan-young
USA	Algae-derived flexible foam, and method of manufacturing the same	US20170066893A1	Bloom Health Holdings LLC
USA	Process for preparing an algal powder containing a reduced content of proteins, and bioplastic composition formulated from such a powder	US20180258231A1	Lavoisier Philippe, Pierre Ronan, Benoit Maud
USA	Algae-derived antimicrobial plastic substrates, and a method of manufacturing the same	US20170183469A1	Falken Robert, Hunt Ryan, Zeller Ashton
USA	Edible cup and method of making the same	US20210204562A1	Chelsea Briganti, Leigh Ann Tucker, Monica Bhatia, Kevin Stanton

**Table 2:** Patents related to bioplastic production using microalgae

Country	Name of patent	Patent	Inventor/s
Australia	Novel biodegradable polymer composition useful for the preparation of biodegradable plastic and a process for the preparation of said composition	AU2007245266A1	Supreethi Sumanam
China	Method for continuously producing hydrogen and polyhydroxyalkanoates by taking blue-green algae as substrate through coupling fermentation	CN101993896A	Yan Qun Zhang, Yibo Tian Xinsheng
China	Algal thermoplastics, thermosets, paper, adsorbants and absorbants	CN104114689A	A. Haring, A. Yeskelayinen, J. Chiuru, C. René, T. Lithier, K. Natinen, J. Pere, S. Sousa, J. Pischoke, A. McKee, J. J. Chernowhouse, A. R. Pavlowski
China	Algal thermoplastics, thermosets, paper, adsorbants and absorbants	CN104114689A	A. Haring, A. Yeskelayinen, J. Chiuru, C. René, T. Lithier, K. Natinen, J. Pere, S. Sousa, J. Pischoke, A. McKee, J. J. Chernowhouse, A. R. Pavlowski
China	Degradable plastic containing algae protein and preparation method of degradable plastic	CN104479369A	Zhu Nianqing, Wang Qing, Chen Haiyan
China	Biobased polyolefin composite material and preparation method therefor	CN105001489A	Wang Qing, Zhu Nianqing, Chen Haiyan
China	Polypropylene composite containing microalgae and preparing method thereof	CN104725727A	Zhu Nianqing, Wang Qing, Chen Haiyan
China	Algae protein/polylactic acid (PLA) biodegradable blending material and preparation method thereof	CN105038167A	Zhu Nianqing, Wang Qing, Chen Haiyan
China	Method for preparing polyolefin algae plastic through microwaves	CN105330931A	Zhou Fuhai, Pan Qi
China	Thermoplastic prepared from algae	CN105331063A	Zhou Fuhai, Pan Qi
China	Blue alga-based compound biological plastic with nano-calcium nutrient enriched and preparation method of biological plastic	CN105907066A	Chen Kexia, Chen Keliang, Chao Jianping
China	Layered nano filler montmorillonite modified cyanobacteria-based compound bioplastic and preparation method thereof	CN105968761A	Chen Kexia, Chen Keliang, Chao Jianping
China	A kind of organic nano bentonite modified cyanophyceae base Biocomposite material and preparation method thereof	CN106117999A	
China	Preparation method of microalgae-based polymer composite thin film	CN105860111A	Shi Bo, Liang Liang, Guo Yongjun, Chen Qiutong
China	A kind of degradable blue-green algae base biological plastics and preparation method thereof	CN107057247A	Jiangsu Academy of Agricultural Sciences
China	The EVA expanded materials and preparation method of a kind of biomass of blue-green algae containing thermoplasticity	CN106867175A	Tang Ailan
China	A kind of green composite foam material and preparation method thereof	CN109679135A	Wang Wei, Zhang Baikai, Jiang Shuai, Hua Tianyu, Wu Quan, Qian Yin Jiangang
China	Green alga environment-friendly sole material and preparation method thereof	CN110591405A	
France	Edible bioplastic from seaweed and the manufacturing technology thereof	WO2014108887A2	Noryawati S, Si. Mulyono
Italy	Process for producing starch from microalgae	WO2017130106A1	Pagnanelli Francesca, Toro Luigi, Di Caprio Fabrizio, Altimari Pietro
Japan	Method for producing sheet material using microalgae	JP2004162209A	Naotaka Fujitani, Hiromi Seki, Akira Morikawa, Yuji Yamaguchi, Hiroyuki Takenaka
Japan	Production method for polyhydroxyalkanoate using only photosynthesis	JP6492011B2	Matsui Minami, Shio Kurihara, Nyokushin Rauhoon, Chun Pin, Sudish Kumar
Japan	Method for producing plastic starting material and related substance from cyanobacteria	WO2015115520A1	Takashi Koyamauchi, Yumi Hirai, Saito Kazuki, Saito Ayuko, Iijima Kuwahara
Japan	Method for producing plastic raw materials and related substances in cyanobacteria	JP5946080B2	Takashi Koyamauchi, Takashi Koyamauchi, Yumi Hirai, Yumi Hirai, Kazuki Saito, Kazuki Saito, Keiji Numata Numata
South Korea	Production of polyhydroxyalkanoates from the saccharified solution of hydrodictyaceae algal biomass	KR101293639B1	Kim Jin-seok, Park Si Jae, Hwang Hyun-jin, Choi Jeong-seop, Lee Seung-hwan, Song Bong-geun
South Korea	Methods for producing organic acids from the saccharified solution of hydrodictyaceae algal biomass	KR20130099475A	Kim Jin Seog, Kim Jin Cheol, Kim Yeong Un, Choi Gyung Ja, Lee Seung Hwan, Kim Seul Ki, Nguyen Mai Cuong, Park Myung Soo
USA	Algae-blended compositions for thermoplastic articles	EP2424937B1	Bo Shi, James H. Wang
USA	Use of marine algae for producing polymers	US20120165490A1	Scott R. Lindell, Christopher M. Reddy
USA	Algal thermoplastics, thermosets, paper, adsorbants and absorbants	US9758757B2	
USA	Methods of bioplastic production	US20130344550A1	Miller Charles, Rahman Asif, Sims Ronald, Sathish Ashik, Anthony Renil,
USA	Polymer compositions comprising algae materials	US20140273169A1	Frederic Scheer, Kelvin T. Okamoto, William E. Kelly
USA	Algae-based bioplastics and methods of making	US20200263125A1	Naohiro Kato
USA	Method to produce a polysaccharide gel by increasing the pH of the polysaccharide	US10907223B2	Mona Mirsiaghi, Eric Sundstrom, Deepti Tanjore, Todd Pray, Rocco L. Mancinelli, David T. Smernoff

**Table 3:** Patents related to bioplastic production using both macroalgae and microalgae

Country	Name of patent	Patent	Inventor/s
Africa	A process for preparing a coating to improve the efficiency and quality of fertilizers	MA40103B1	Mustapha Benmoussa
South Korea	Environmental-friendly polymer composition and method of fabricating the same	KR102160900B1	Kim Byung-yong
South Korea	Composition for manufacturing biodegradable plastic seaweed farming nets containing seaweed fertilizer ingredients	KR102393864B1	Cho Cheon-rae
USA	Algae-blended compositions without plasticizers	AU2016243338B2	Michael Lawrence Gross, Ryan Webster HUNT, Bo Shi, Mark Ashton Zeller
USA	Algae-blended thermoplastic compositions	US20200283600A1	Bo Shi, Michael Lawrence Gross, Ryan Webster Hunt, Mark Ashton Zeller
USA	Algae thermoplastic composition and process of making	WO2020237232A1	Mark Ashton Zeller, Ryan Hunt

A total of 53 patents using algae for bioplastic production have been published since 2011 (Fig. 6b). The published patents were significantly lower than that of the corresponding number of published papers, indicating a lower level of transformation of the basic and/or applied research into practical applications. The majority of the patents were filed in 2011 and 2015–2016, with eight published patents in 2016 and six in 2020 (Tables S1-3). In later years, the number of patents was lower, and in 2016, it again increased, and a spike occurred in 2020 that shows the recent increase in interest in this field. This is consistent with the rise in global awareness of the worldwide negative impact of conventional plastics and the need to reduce reliance on these materials. The tendency to file patent applications differs significantly across technical fields and varies from company to company. Particularly with regard to the production process, companies tend to avoid patenting their discoveries (Elvers et al. 2016).

Geographically, patent filings (46%) by China far exceeded those from the rest of the world. The majority of the other patenting activity appears to be in the USA and South Korea showing 34%, ~ 27%, and ~ 20% of patents arising from those countries respectively. Since these countries are major economically developed or developing countries/regions in the world, investments in bioplastic sources are driven by the government.

**Fig. 5:**
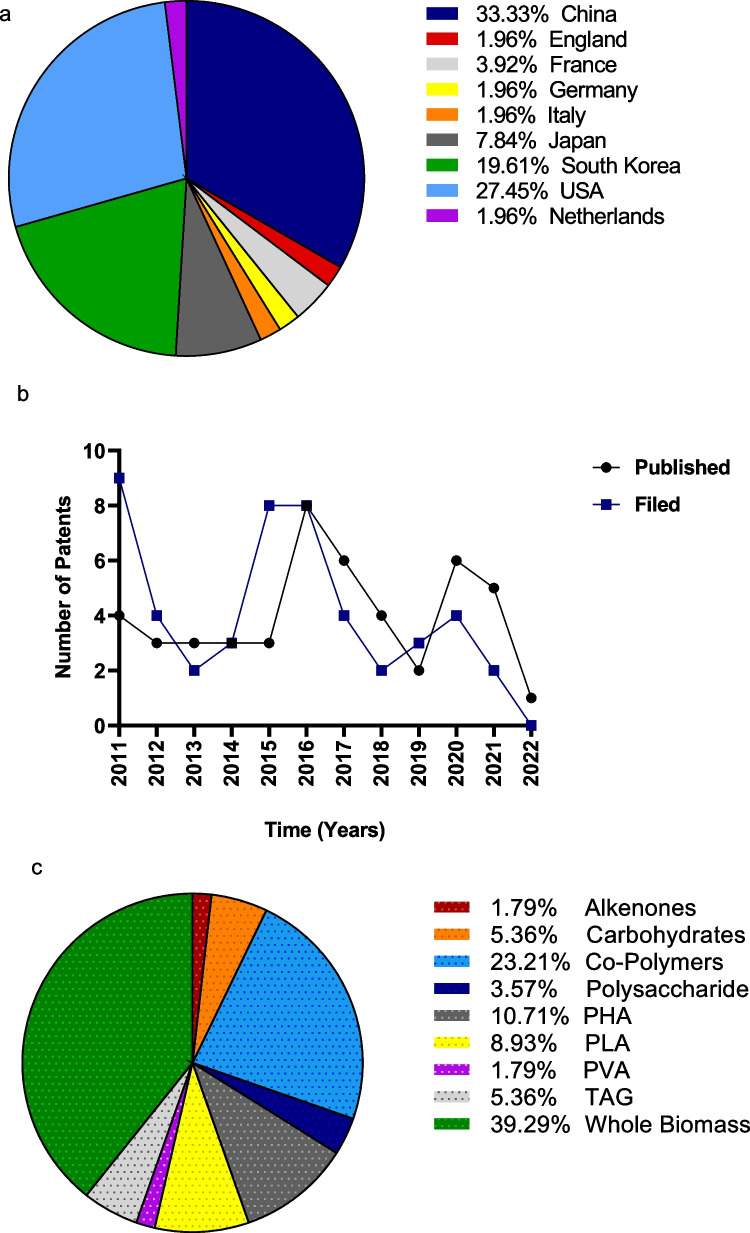
**a** Worldwide distribution of patents, **b** number of patents filed and published between the years 2011–2022, **c** patents related to the type of product used for bioplastic production

Although the publication outputs have been limited in China, they are leading, with regard to new intellectual property (Supplementary data Table s1). A large number of patents are possible since the Chinese government currently subsidizes patent applications as part of its overall domestic industrial policy agenda. Moreover, the high number of patent filings from China highlights the country’s commitment to innovation and addressing environmental challenges associated with plastic pollution (Liu et al. 2022b; Rosenboom et al 2022). China is one of the countries that has invested a large amount of money and research in the area of algal biotechnology. Furthermore, they declared that they will no longer be receiving waste from other countries for recycling after December 2017 (Joyce 2019). The high patent activity in the USA is mainly because of large private and public investment in both academic and industrial R&D. However, based on the publication data (Fig. 5a), research activities, and several large-scale innovations, there will be a potential change towards greater international patent filing in Europe. Arora et al. (2023) stated that marketing strategies employed in Germany to increase public awareness on this topic proved positive and successful. Such marketing initiatives and awareness should hence be implemented at a larger scale globally.

Previously, the patenting process has been reported to be slow, whereby it takes 3–4 years to decide whether a patent is granted or not (Tidwell and Liotta 2012). However, recently, several patent offices have allowed the acceleration of patent applications for green technologies such as bioplastics. Accelerating the patent application process reduces the time to grant the patent to under a year. The countries offer options for accelerating environmentally friendly technology, including the UK, Japan, China, South Korea, Australia, Israel, Canada, Brazil, and Taiwan.

Patent claims can broadly be grouped into two types: products and activities such as manufacturing methods or specific uses of a product. The majority of the patents seen in Fig. 5 deal with the process of producing plastic from algae. Most patents (~ 39%) use whole algal biomass. This is owing to the economics; whole microalgae without any product extraction method blended with other polymers have a lower overall cost (Kim et al. 2022). It is worth mentioning that the application of whole biomass for plastic production often results in a relatively lower quality of the final products thus many hindered its commercialization. A similar trend to the publications (Fig. 4) is seen with the patents, whereby plastics are primarily produced using PHAs and co-polymers. Although there is a worldwide increase in research activities, there are still only a few granted patents that are available on the market. Findings highlight the significance and necessity for R&D investments within industries as well as cooperation with academia in order to improve bioplastic properties, enhance the quality of algae-based bioplastics, and industrialize new techniques for bioplastics production (Chia et al. 2020).

## Current research scale-up projects and initiatives

An excellent recognition of the ability of algae as a feedstock for biobased plastics is evident in large-scale projects and pilot-scale investigations. These projects aim to deliver sustainable strategies and solutions for biobased plastic production and promote a circular economy (Supplementary data Table S2).

Recent advancements in algal bioplastics published by the World Economic Forum & EcoWatch (2023) highlighted the potential of using *Spirulina* for bioplastic production. The research conducted at the University of Washington demonstrated that these algal bioplastics biodegrade naturally and relatively quickly, as well as they are also recyclable through mechanical recycling processes. Despite some challenges, such as brittleness and water sensitivity, researchers have made significant improvements in the stiffness and strength of these bioplastics by adjusting processing conditions like temperature, pressure, and time.

The Nenu2PHAr project is a 5-year project that started in 2020, financed by the H2020 European program aimed to develop a viable alternative to existing petrochemical-based plastics that is sustainable and biodegradable (Nenu2PHAr 2020). This project brings together 17 partners (5 large industries, 6 small- and medium-sized enterprises (SMEs), 5 Research Technology Organisations (RTOs), and 1 cluster), leaders in the different fields of research, from biomass development to formulation of biopolymer-up to plastic processes (Europa n.d.). Eight PHA-based products are expected to be developed and benchmarked against their fossil-based counterparts. Microalgal biomass will be used as the primary feedstock for PHA-accumulating bacteria. This microalgal biomass is cultivated under specific conditions to trigger an accumulation of starch. Then, this biobased substrate will be used as a carbon feedstock for bacteria cultivation to accumulate the PHA polymer (Alvarez et al. 2021; Geerinck and Schueren 2021).

Another interesting 2-year project supported by the H2020 European program was the Seaweeds From Sustainable Aquaculture As Feedstock For Biodegradable Bioplastics (SEABIOPLUS) project. This project commenced in October 2013 and was completed in September 2015 in Ireland. Two end products were developed, PLA from *Ulva lactuca* and polysaccharide films from *Gracilaria vermiculophylla.* The production of seaweed in SEABIOPLAS was done in integrated multitrophic aquaculture (IMTA) systems, i.e., seawater used was nutrient-enriched, whereby nutrient wastes from fish farms were recycled. This study was one of the first analyses of the environmental performance of seaweed in polymer applications.

The study identified that electricity consumption was a major drawback. For lactic acid production, *U. lactuca* cultivation required high energy as well as enzymes used in the hydrolysis whereas, for polysaccharide film production, most of the environmental impact was due to electricity consumption during the extraction of polysaccharides from the dried *G. vermiculophylla*. This work was useful in demonstrating that the chemical content of seaweed biomass can be effectively manipulated in a land-based cultivation system. Helmes et al. (2018) also reported that the experimental-based seaweed scenario score turned out to be more than ten times higher than the reference maize scenario. It identified differences in electricity consumption as the main reason for the low performance of seaweed production. The data showed that majority (99%) of electricity consumption happened during the cultivation stage, due to water pumping and aeration of the seaweed basins. Concurrently, the outcomes of the optimization scenario suggest that improvements in material efficiency during cultivation and purification processes could significantly enhance environmental outcomes. The researchers expect that future upscaling will contribute to higher efficiency, demonstrating another substantial improvement.

The MakPak project (sustainable packaging solutions from marine macroalgae for the food sector) (MakPak 2018) was a collaboration between the Institute Alfred-Wegener Institute (AWI) and Nordsee GmbH (Lomartire et al. 2022). The aim was to sustainably produce a disposable and/or edible packaging solution for food items. The packaging consisted exclusively of raw materials from marine macroalgae. The prototype is expected to be optimized for manufacture on an industrial scale. Nordsee anticipates being able to offer the new packaging on a commercial basis in two to three years. The follow-up project Mak-Pak Scale-Up commenced in 2021. The goal was to scale up and optimize the production of the macroalgae that have the potential to be used to create sustainable, biodegradable, and/or edible macroalgae-based packaging material for the food industry.

The Sustainable PoLymers from Algae Sugars and Hydrocarbons (SPLASH) project (2012–2017) (http://eu-splash.eu/) comprised 20 partners, SMEs, large enterprises universities, and research institutes. Microalga *Botryococcus braunii* was used for the production and recovery of hydrocarbons and exopolysaccharides and their conversion to renewable polymers such as bioplastics. This project demonstrated a proof of concept for an industrial process of exopolysaccharides recovery. They applied the milking process to extract 12% of the exopolysaccharides daily without compromising the viability of the cultures. From the analysis, it was reported that expenditures were the cultivation of microalgae in the photo-bioreactors followed by the milking/separation step.

In 2017, designers Eric Klarenbeek and Maartje Dros of Studio Klarenbeek & Dros initiated a project with Atelier Luma (France) called Algae Lab, a bio-laboratory that researches the potential of growing micro- and macroalgae exploiting their applications. Algae Lab converted various algae as binders, fibers, or pigments for biopolymers to 3D-print objects from shampoo bottles to tableware or rubbish bins (AlgaeLab 2016). In 2019, Atelier LUMA collaborated with furniture manufacturer Vitra to produce the Algues modules, designed by the Bouroullec brothers. This demonstrates the diversity and wide range of applications of algae bioplastics.

International research networks provide platforms for knowledge exchange for researchers and entrepreneurs. The development of these large-scale algae bioplastic projects and the involvement of government in Europe and other regions signify a commitment to transition to environmentally friendly biomaterials. Large-scale projects provide a platform for innovation and research in the field. They enable the investigation of effective cultivation methods, optimized extraction processes, and the development of bioplastic with enhanced properties.

## Global developments in the bioplastic industry and applications of commercially available algae-based bioplastics

**Fig. 6:**
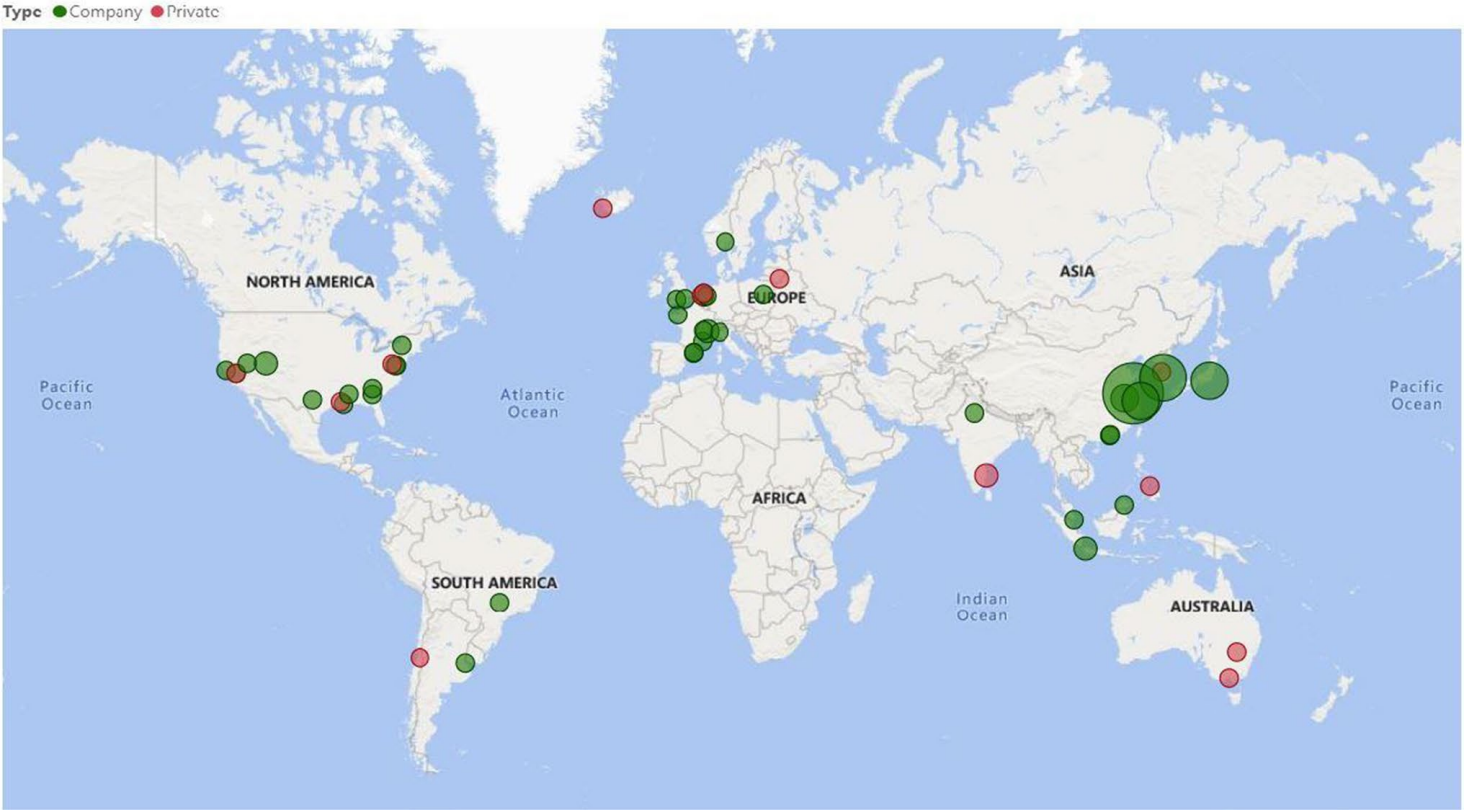
Global map depicting commercial companies (green circles) and private businesses (red circles) that are utilizing algae/cyanobacteria to produce biobased product

It is evident from the map (Fig. 6) that there is a current global upsurge and advancement in utilizing algae/cyanobacteria for bioplastic applications. Various algal-based bioplastic-producing plants are situated in different parts of the world (Table S3). Our research suggests that there are approximately 81 entities worldwide that are currently producing biopolymers/bioplastics from algal/cyanobacterial species. Most of these entities are commercial companies (green circles), while 13 out of the 81 are small, private businesses (red circles). China appears to have the greatest number of establishments (24.7%), followed by the USA (18.5%) and then South Korea (9.9%). It has been estimated that in China, approximately 40% of used plastics end up as litter or in landfills, and the country is also responsible for a quarter of the global plastic waste that pollutes oceans.

Owing to this China’s top economic planning body, the National Development and Reform Commission introduced a policy aimed at limiting the use of non-biodegradable plastic. Japan (6.2%) also has a few commercial companies that are currently focused on biomass (algae/cyanobacteria) plastics. The goal of the Japanese government is to use biomass plastic material for energy generation through incineration, and they also aim to produce 2 million tons of all bioplastics a year by 2030.

The South Korean government inaugurated a project in June 2022 to develop technologies to valorize *Sargassum horneri*, to produce itaconic acid, 3-hydroxypropionic acid, and lactic acid used to manufacture bioplastics. From the map, it appears that to date, Australia does not have any commercial companies that are invested in this endeavor. They do, however, have some smaller, private initiatives (2.5%) that are underway. Compared with the rest of the world, research suggests that Africa lags in the utilization of algal species for bioplastic production. There are currently no commercial/private facilities that are working on this endeavor within Africa. Most of Africa, especially the southern parts, has great potential as a competitive producer of algal-based bioplastics due to the optimal climatic conditions for algal growth, including plenty of sunlight and warm temperatures as well as vast coastal resources. Owing to this scenario and with unified efforts and investments, Africa has the resources and ability to establish a significant algal bioplastic industry.

Based on a recent report by Rosenboom et al. (2022), several countries have adopted new laws that have banned plastic products like consumer plastic bags. Of the 192 countries, 127 (72%), have some type of ban/restriction on the manufacture, importation, and distribution of plastic bags. Additionally, some countries have implemented some type of tax on single-use plastics, either as a special environmental tax, waste disposal fees, or in the form of higher excise taxes (Ghaffar et al. 2022; OECD 2013; United Nations Environment Program 2018). Several countries have also committed to the development of natural plastic alternatives to support UN SDGs by reducing the dependence on conventional plastics and promoting a shift towards green alternatives contributing to climate change mitigation (Moshood et al. 2021). Support from governments is evident in policies that incentivize R&D in the field of algal bioplastics, funding initiatives to scale up production, and offering regulatory frameworks that encourage the adoption of these environmentally friendly materials.

As seen in Fig. 6, many European countries are presently involved in the production of biopolymers/bioplastics from algal/cyanobacterial sources. A report by Pira International stated that Europe held the largest regional market for bioplastics packaging in 2010. This could be attributed to the strict European government policies pertaining to recycling maintenance and composting infrastructure as well as responsible retail and consumer attitudes towards sustainable packaging. In 2012, a report (Science for Environmental Policy 2012) by the European Commission DG ENV stated that the production of bioplastics using algae could represent a more cost-effective method on a commercial scale as opposed to current techniques. In December 2019, the European Commission published the “European Green Deal” (EGD) initiative aimed to increase the efficient use of resources by moving to a clean, circular economy and stopping climate change, reverting biodiversity loss, and decreasing pollution (Di Bartolo et al. 2021; Siddi 2020).

Applications of algae-based bioplastics vary from packaging, food, electronics, automotive, medical, agriculture, and toys to textiles. Currently, there are several commercially available algae-based bioplastics as well as some products manufactured by designers or researchers on a small scale (data available in supplementary Table 2). At present, packaging for food and beverage, healthcare, and cosmetics continues to be the largest market for bioplastics. The companies Notpla, London (Notpla 2015), and Evoware, Indonesia (Evoware 2016), are producing packaging from algae. Notpla manufactures a wide range of products from cardboard coating, sauce sachets, and take-away boxes, to edible single-use packaging that biodegrades within 4 to 6 weeks. Evoware uses the algae to package confectionery, tea, or spices.

Additionally, Cereplast, Inc., USA (Cereplast 2008), designs and manufactures sustainable bioplastics from algae which are used as substitutes for traditional plastics in all major converting processes such as injection molding, thermoforming, blow molding, and extrusions required in industries such as automotive and consumer electronics. They offer algae resins with high biobased content and compostability suitable for single-use applications in the food service industry. Algix, USA (Algix 2021), produces a cost-competitive, biobased, and thermoformable resin (algae-based foams) that can be used in everything from shoes to outerwear, to everyday household items (Khyalia et al. 2022). Checkpoint is another company using microalgae to produce performance materials and has already worked with WNDR Alpine, USA (WNDR Alpine 2016), to replace conventional plastics used in skies with algae-based performance material. Austeja Platukyte developed biodegradable lightweight, waterproof, strong, and durable materials from algae, consisting of agar from algae and coated calcium carbonate, that could replace petroleum-based plastics (Chia et al. 2020). A Korean research group led by Professor Park Jin-byung from Ewha Womans University has developed a technology to manufacture eco-friendly bioplastics using green algae from the ocean. The team succeeded in producing an environmentally friendly carboxylic acid using fat and fatty acid that can be used to manufacture high-performance engineering plastics.

Zerocircle, India (Zerocircle 2020), uses carbohydrates extracted from macroalgae to make a flexible plastic film for packaging. Furthermore, Chile-based designer Margarita Talep has created single-use packaging using raw materials, i.e., polymers and agar extracted from red algae. Similarly, Ari Jónsson used red algae to create a sustainable alternative bottle to the conventional plastic bottle. While the bottle maintains its shape when filled with water, it starts decomposing once empty. The liquids stored in the bottle are safe to drink, and the consumers even have the option to consume the bottle itself. Sway, California (Sway 2018), also uses macroalgae to manufacture compostable thin-film plastic alternatives.

## Future prospects

The prospects of algae-based bioplastics hold promising potential within the framework of the circular economy, a sustainable solution that has come to the forefront of many discussions in recent years. Aligning with the circular economy approach, algal bioplastics could offer a sustainable closed-loop solution. The circular economy model encourages longer market circulation times for raw materials before disposal. The end-of-life cycle for algae-based bioplastic products could involve transformative processes like composting, followed by potential applications as peat substitutes, and agricultural films as they decompose in soil effectively terminating the bioplastic life cycle (Devadas et al. 2021; Torres et al. 2015).

Algae-based plastic products have a distinctive advantage over other bioplastics since algal biomass can be cultivated throughout the year and is also sustainably sourced. Using wastewater resources instead of chemical-based components could substantially cut down cultivation medium costs (Devadas et al. 2021; Karan et al. 2019; Leong et al. 2019). However, there are some additional bottlenecks affecting the production process and cultivating microalgae to high cell density due to nutrient limitations, light availability, and potential contamination issues. Researchers have focused on overcoming these challenges, by employing genetic engineering and optimizing factors influencing algae cultivation, such as nutrient availability, light exposure, and temperature. Lim et al. (2022) reported that the integration of IoT and AI introduces smart farming practices to enhance the productivity and sustainability of algal cultivation, potentially serving as a precursor for improved control and monitoring using real-time data and predictive modeling. These breakthroughs represent a critical step forward in advancing the feasibility and scalability of algae-based bioplastics, contributing to the sustainable evolution of biobased materials in a manner that aligns with the principles of environmental responsibility and circular economy practices.

The biodegradability of algae-based bioplastics and lower cost if a closed resource cycle and cascades approach for algal cultivation is applied align with environmental safety and economic considerations. Algae have a conversion efficiency of ± 90% from raw material to bioplastic and the capacity of algae to grow in high CO_2_ concentrations which further highlights their environmental benefits (Karan et al. 2019; Devadas et al. 2021). Furthermore, extracting monomers or polymers from algae waste residues for the production of consumer-grade bioplastics presents a sustainable solution to waste disposal issues (Chia et al. 2020; Torres et al. 2015).

In the future, algal bioplastic production could involve the utilization of gene-editing tools like CRISPR to enhance metabolite production within algal species. This genetic engineering approach, along with nutrient-deficient conditions, holds the potential for upscaling algal bioplastic production (Selvaraj et al. 2021). Most studies conducted on algae bioplastics are being done at a laboratory scale, with an increasing number of large-scale projects, and the upsurge in scientific attention emphasizes the growing commercial potential of algal-based bioplastics. However, it is crucial to balance long-term investments in R&D with an emphasis on minimizing production costs for sustained market growth.

Although PLA is the most promising polymer for bioplastic production, the technological maturity of PLA requires cautious long-term investment decisions. PLA can be biodegraded efficiently in industrial composting facilities with controlled conditions such as high temperatures and high humidity levels (Kalita et al. 2021). Future R&D directions should emphasize minimizing production costs to increase market growth. In contrast, PHA technologies are in a growth phase, with significant potential due to their thermoplastic processibility, hydrophobicity, high degree of crystallinity, optical purity, gas barrier properties, and high melting temperature (Sharma et al 2021). The path to global commercialization of algal-based bioplastics requires significant monetary investments, knowledge transfer, and collaborative efforts to enhance competitiveness and scalability. Nevertheless, several countries are making significant efforts to promote bioplastic production, though the costs of scale-up are a limitation. The successful combination of algae and other biobased materials could yield bioplastics with similar qualities to conventional plastics and allow the product to transition from the research stage to the development and production stage. Further research into the development of bioplastic production processes, techno-economic analyses, and comprehensive life cycle assessments of algal bioplastics is imperative. A better understanding of the “gate to grave” approach of algal bioplastics would aid in filling in the gaps for further development of this technology (Organization for Economic Co-operation and Development 2013). Public awareness and perception of algal-based bioplastics also need to be elevated to address the global plastic challenge. The potential for algae-based bioplastics is growing rapidly, driven by ongoing technological advancements, investments, and a commitment to the environment and a sustainable future.

## Conclusion

This review provides a comprehensive and informative exploration of technology trends in algal-based bioplastics, providing insights for researchers and industry stakeholders involved in advancing the field of sustainable materials. This review highlights the progress within the field of algal bioplastics in the past decade. Aside from summarizing progress made at a research level, novel insights into companies worldwide that are currently manufacturing bioplastic components from algal biomass are also included. This article also serves to emphasize the challenges and further research required to increase the commercial feasibility of this product in the future. Within the last decade, there has been mounting attention from governments, researchers, and the scientific community which has led to an increase in the number of scientific publications, research projects, and patents in this area. The plethora of scientific evidence presented in this article suggests that algal bioplastics have the potential to be an environmentally friendly and biodegradable option that could help reduce dependency on traditional plastics.

### Supplementary Information

Below is the link to the electronic supplementary material.

Supplementary file1 (DOCX 23 KB)

Supplementary file2 (DOCX 16 KB)

Supplementary file3 (DOCX 30 KB)

## Data Availability

Not applicable.
