# Supplementary material for: Algal-based bioplastics: global trends in applied research, technologies, and commercialization
Source: Environ Sci Pollut Res Int. 2024 May 24;31(26):38022–44. doi: 10.1007/s11356-024-33644-9 (PMC11189328; doi:10.1007/s11356-024-33644-9)
Supplement: Supplementary file 1 — Supplementary file1 (DOCX 23 KB) [file 11356_2024_33644_MOESM1_ESM.docx]

Supplementary data Table S1

| **Name of Company / Individual** | **Collaboration** | **Technology** | **Type of organism** | **Type of Bioplastic** | **End Product** | **Company Location** | **Year Founded** |
| --- | --- | --- | --- | --- | --- | --- | --- |
| Corbion | accquire TerraVia |  |  | Polylactic acid (PLA) |  | Netherlands | 1919 |
| International Technology Management Associates Ltd |  | A foamed and stabilized algal fiber matrix having substantial dimensional stability. | Filamentous green algae such as Cladophora glomerata | Polysaccharide, cellulose & plastcizer | molded packings or foamed particles packings | Chantilly, US | 1993 |
| National Institute of Ocean Technology (NIOT) |  | macro-algae incorporated with the plasticiser polyethylene glycol (PEG)-3000 to v | Red algae Kappaphycus alvarezii |  | bioplastic films | Chennai, India | 1993 |
| Cereplast, Inc (Algaeplast) |  | Company commercialized small quantities of Biopropylene 109D, a compound with 20% post-industrial algae biomatter. Algaeplast has been created to develop a new monomer and polymers made from algae | Algae | Polymers | Biopropylene (R) 109D, polymers | Nevada, US | 2008 |
| Photanol |  | The cyanobacteria’s metabolic pathways are enhanced to produce monomers used for different plastics, thereafterrenewable chemistry is used to create circular plastics | Cyanobacteria |  | Circular plastics | Amsterdam, Netherlands | 2008 |
| Algix | University of Georgia and technical innovations licensed from Kimberly-Clark, | Blends aquatic feedstocks (microalgae, cyanobacteria etc) with commercial polymersthermoplastic polymeric composition that includes at least one kind of algae or a blend of at least one kind of algae and a plant polymer. | Green algae and cyanobacteria (Nannochloropsis and Spirulina) | Thermoplastic polyester | Formed into bioplastic articles, sheets & films, including injection molded parts, injection molding applications. | Mississippi, US | 2010 |
| Algo Pack |  | has developed a unique, innovative process to transform algae into rigid biomaterials. | Brown macroalgae (*Sargassum* and *Laminaria*) |  | Flower pots, algae blend product, pens, | Saint-Malo, France | 2010 |
| Eranova |  | Extracts strach from green macroalgae for use as a resource for the production of bio-based, recyclable, compostable resins. The algae is deprived of nutrients which reduces the protein content and in turn increases the self-production of starch (the raw material for bioplastic pellets) | Green macroalgae |  |  | France | 2012 |
| Studio Nienke Hoogvliet |  |  | Macroalgae |  | Yarn and bioplastic bowls | The Hague, The Netherlands | 2013 |
| Notpla (Skipping Rocks Lab) |  | Flexible, 100% biodegradable & revolutionary material (edible containers) made from brown macroalgae | Brown macroalgae |  | edible packaging: Oohoo capsules (edible) & seaweed-lined greaseproof takeaway boxes | London, UK | 2014 |
| AlgaeLab (Eric Klarenbeek and Maartje Dros) | Danvos, Wageningen University, Biobased Lab in Breda |  | Salga Seaweeds |  | liquid polymer to 3D-print objects from shampoo bottles to tableware or rubbish bins | Zaandam, The Netherlands | 2014 |
| Loliware (Chelsea Briganti ) |  | Production of hypercompostable, edible materials derived from red macroalgae (used Carrageenan and Agar from algae) | Red marcoalgae |  | Single-use items like drinking straws, cups, etc. | New York, US | 2015 |
| [RWDC Industries](https://www.rwdc-industries.com/) | Kimberly-Clark | Ferments microalgae from food waste to produce fully biodegradable material (Solon) which can replace traditional petroleum-derived plastics. Solon is made from polyhydroxyalkanoates (polymer) | Microalgae | PHA | Drinking straws, bottles, cutlery, paper coatings, and fibres (that can be used to produce items such as wet wipesand diapers) | Singapore | 2015 |
| Evoware (Evo & Co.) |  | Macroalgae-based packaging that dissolves in hot water and can be consumed | Macroalgae |  | Edible cups (Ello Jello), food wrappings (for protein bars, burgers, waffles etc.) & sachets (containing instant coffee, tea or flavouring for noodles etc.) | Jakarta, Indonesia | 2016 |
| Atelier LUMA | Maartje Dros, Erik Klarenbeek | Starch from cyanobacteria and algae blended with another biopolymer that can be used in 3D printing | Microalgae, Macroalgae and Cyanobacteria | Polymers | vessels | [Arles, France](https://maps.google.com/?q=Atelier%20LUMA%20%0D%0AParc%20des%20Ateliers%2C%20%0D%0A53%20chemin%20des%20Minimes%2C%20%0D%0A13200%20Arles%2C%20France) | 2016 |
| Austeja Platukyte |  | Biodegradable packaging made from algae agar, calcium carbonate, water and a and a vegetable based emulsifying mix | Algae |  | bowl like structures | Vilnius, Lithuania | 2016 |
| Ari Jónsson |  | Algae agar is combined with water to form a jelly-like substance.The substance is then heated and decanted into a bottle-shaped mould where it chills in icy water before it solidifies in a refrigerator for a few minutes.Once set, the jelly can be removed from the mould as a solid water bottle. | Red Algae |  | Algae-based water bottle that decomposes when empty | Reykjavík, Iceland | 2016 |
| Park Jin-byung (Marine Biomaterials Research Center,Ewha Womans University) |  | Fat and fatty acid extracted from algae and used to produce a carboxylic acid, which is a material that can be used to manufacture high-performance engineering plastics | Green algae or microalgae | Carboxylic acid |  | Korea | 2016 |
| Algaene |  | Genetically modified technology |  |  | biodegradable medical devices, children's toys, | Guangdong, China | 2017 |
| AlgoTek |  |  | Macroalgae |  | plastic film that can be dissolved in water: Cups, FIlm, Takeaway | Portland, Oregon | 2017 |
| AlgiKnit |  |  | Macroalgae (Kelp) |  | Yarn | New York | 2017 |
| Sway |  |  | Macroalgae |  | bags | Berkeley, California | 2018 |
| B'zeos | Nestle |  | Macroalgae |  | straws and films | Norway | 2018 |
| SoluBlue |  |  | Macroalgae |  | bioplastic films and bags | London, UK | 2018 |
| Oimo |  | Oimo Material  is generated by  heating the natural molecules present n the algae extracts to produce a chemical reaction that obtains a series of molecular chains that adhere to create Oimo bioplastic |  |  |  | Barcelona, Spain | 2019 |
| Margarita Talep |  | Bioplastic made up of a polymer, a plasticiser and an additive. The agar mixture is boiled to around 80° Celsius to make a material that bears a close resemblance to thin plastic. Once melted, the molten liquid drops to below 20° Celsius | Red algae |  | single-use packaging | Santiago, Chile | 2019 |
| OTHER MATTER (Jessie French) |  | Uses processed red algae extract or microalgae with mixed pigments. Mixes up the recipe for that day, and then it goes into the saucepan and cooks for about 45 minutes.From there, the designs are handmade and poured. | Red algae and microalgae |  | tableware vessels, bowls, cups, and plates, lighting fixtures | Melbourne, Australia | 2020 |
| Zerocircle |  | Carbohydrates are extracted, gelatinized and processed to make a flexible plastic film roll. | Macroalgae |  | bioplastic films | Guragaon, India | 2020 |
| Marea |  |  | Macroalgae | Biopolymers | packaging | Iceland | 2020 |
| Kelpi | University of Bath |  |  |  | packaging | UK | 2021 |
| WNDR Alpine's | Checkerspot | [Skis produced from Aspen wood, which is reinforced through a core made of an algae-derived hard foam, similar to high-density polyurethane. unprecedented degree of flexibility in the design and development of skis](https://www.dezeen.com/tag/polyurethane/) | Microalgae | Polyurethanes. | skis | Utah, US | 2016 |
| AMAN |  | Agar Plasticity as a plastic alternative | Red macroalgae |  | packaging, sheets for wrapping, | Japan | 2000 |
| AlgaePARC |  | Research into cellular processes, strain development, cultivation, optimisation, scale up, biorefinery and product development and chain analys | Algae and cyanobacteria |  | Sustainable Packaging | Wageningen University, Netherlands | |
| Microalgae LLC (Naohiro Kato) |  |  | Microalgae |  | biodegradable Mardi Gras beads | Baton Rouge, Louisiana |  |
| Alternative Plastics | Eranova |  |  |  |  |  |  |
| Dr. Pia Winberg |  | Sulfated polysaccharides. Alginate | Green macroalgae (Ulva) |  | cotton replacement (fabric) | New South Wales, Australia |  |
| PARLEY AIR |  | PHA |  |  |  | globally |  |
| Rhodomaxx |  |  | macroalge (Kappaphycus alvarezii) |  |  | Malaysia |  |
