# Supplementary material for: Algal-based bioplastics: global trends in applied research, technologies, and commercialization
Source: Environ Sci Pollut Res Int. 2024 May 24;31(26):38022–44. doi: 10.1007/s11356-024-33644-9 (PMC11189328; doi:10.1007/s11356-024-33644-9)
Supplement: Supplementary file 2 — Supplementary file2 (DOCX 16 KB) [file 11356_2024_33644_MOESM2_ESM.docx]

Supplementary Table S2: Pilot Scale projects

| **Name of Project** | **Start-end date** | **Collaborating Partners** | **Organism** | **Type of Bioplastic / End-product** | **Project fund amount** | **Funders** | **Link** | **Location** |
| --- | --- | --- | --- | --- | --- | --- | --- | --- |
| NENU2PHAR | 2020-2024 | The French Alternative Energies and Atomic Energy Commission, CEA; Innovation Plasturgie Composites, IPC; Université de Bretagne Sud; Association Industries et Agroressources; Elixance Masterbatches; Danone Research SAS; Sofradim Production SASU (France); Instituto Tecnologico del Embalaje, Transporte y Logistica Itene;Lomartov SL (Spain), Zero Emissions Engineering BV (Netherlands); Celabor SCRL; Centexbel; IFG Exelto (Belgium); Kaj Zoo (Poland); Bio-Mi Drustvo S Ogranicenom Odgovornoscu za Proizvodnju, Istrazivanjei Razvoj (Croatia); Biotrend-Inovacao e Engenharia em Biotecnologia SA (Portugal) |  | PHA | Total Budget: €6395081,25 BBI JU contribution:€4,983,169.87 | Bio-Based Industries Joint Undertaking (BBI-JU) under the European Union’s Horizon 2020 research and innovation programme. | https://www.bbi.europa.eu/projects/nenu2phar | France, Spain, Belgium, Poland and Portugal |
| MakPak project (Sustainable packaging solutions from marine macro algea for the food sector ) | 2018-2020 | NORDSEE; University of Applied Sciences Bremerhaven;  Alfred-Wegener Institute | marine macroalgae | compostable and edible pack |  | Federal Ministry of Food and Agriculture | <https://www.awi.de/en/science/special-groups/aquaculture/marine-aquaculture/research/mak-pak.html> |  |
| Mak-Pak Scale-Up “Industrial implementation of a production process for a sustainable packaging concept made from macroalgae for the food sector” | 2021-2023 |  | macroalgae |  |  |  |  | based in Hamburg/Germany |
| SEAweed (the SEA stands for Southeast Asia) Tech | 2021-2023 | Coast 4C | macroalgae |  | $840,000 | International philanthropic organisation Julius Baer Foundation, based in Switzerland. | https://www.uts.edu.au/partners-and-community/initiatives/uts-sustainability/sustainable-development-goals/partnerships-goals/seaweed-tech-bio-plastic-manufacture | Philippines, |
| PlastiSea | 2020-2023 | SINTEF (Norway) Seaweed Energy Solutions (Norway) B'ZEOS (Norwa) AITIIP TECHNOLOGICAL CENTRE (Spain) Aalborg University (Denmark) KTH Royal Institute of Technology (Sweden) | macroalgae |  | € 25 000 000,00 | BlueBio | https://www.sintef.no/en/projects/2020/plastisea-novel-enhanced-bioplastics-from-sustainable-processing-of-seaweed/ |  |
| BIOPAL | 2003-2006 |  |  |  |  |  | https://cordis.europa.eu/project/id/QLK5-CT-2002-02431 |  |
| European (FP7) Sustainable PoLymers from Algae Sugars and Hydrocarbons | 2012-2017 |  | Botryococcus braunii |  | € 11 929 616,80 | STICHTING DIENST LANDBOUWKUNDIG ONDERZOEK | https://cordis.europa.eu/project/id/311956/reporting |  |
| Development of bio-plastic production technologies from microalgae | 2012- 2016 | AlgaePARC, Wageningen UR Ewha Womans (University in Seoul) ,local commercial partner Kolon Industries | *Botryococcus braunii* | hydrocarbons and carbohydrates |  |  |  |  |
