# Supplementary material for: Algal-based bioplastics: global trends in applied research, technologies, and commercialization
Source: Environ Sci Pollut Res Int. 2024 May 24;31(26):38022–44. doi: 10.1007/s11356-024-33644-9 (PMC11189328; doi:10.1007/s11356-024-33644-9)
Supplement: Supplementary file 3 — Supplementary file3 (DOCX 30 KB) [file 11356_2024_33644_MOESM3_ESM.docx]

**Supplementary Table 3: Worldwide Algae-based bioplastics patents from 2012- 2022**

|  | **Country** | **Name of patent** | **Patent** | **Type** | **Strain/s** | **Inventor/s** | **Assignee** | **Date Filed** | **Date published** | **Granted** | **Source** |
| --- | --- | --- | --- | --- | --- | --- | --- | --- | --- | --- | --- |
| 1 | China | Method for continuously producing hydrogen and polyhydroxyalkanoates by taking blue-green algae as substrate through coupling fermentation | CN101993896A | Micro | Cyanobacteria | Yan Qun Zhang Yibo Tian Xinsheng | Jiangnan University | 2010 | 2012 | pending | PHA |
| 2 | China | Degradable plastic containing algae protein and preparation method of degradable plastic | CN104479369A | Micro | Cyanobacteria (*Microcystis aeruginosa, Anabena, Spirulina*) | Zhu Nianqing Wang Qing Chen Haiyan | Wuxi Taihu Lake Blue Algae Resources Application Technology Research Institute Co Ltd | 2014 | 2016 | 2016 | Polysaccharide |
| 3 | China | Edible fully-biodegraded seaweed plastic wrap and preparation method thereof | CN104231297A | Macro | Macroalgae | Xu Jiachao Hu Yiming Liang Yunbo Guo Liling | Beijing Gerui Zhibo Ecological Science & Technology Research Institute, Ocean University of China | 2014 | 2017 | 2017 | whole biomass |
| 4 | China | Bio-based polyolefin composite material and preparation method therefor | CN105001489A | Micro | Cyanobacteria | Wang Qing Zhu Nianqing Chen Haiyan | Wuxi Taihu Lake Blue Algae Resources Application Technology Research Institute Co Ltd | 2015 | 2015 | pending | Polymer |
| 5 | China | Polypropylene composite containing microalgae and preparing method thereof | CN104725727A | Micro | Microalgae | Zhu Nianqing Wang Qing Chen Haiyan | Wuxi Taihu Lake Blue Algae Resources Application Technology Research Institute Co Ltd | 2015 | 2015 | pending | whole biomass |
| 6 | China | Kelp powder biodegradable plastic and preparation method thereof | CN104829873A | Macro | Macroalgae | Hefei Ring Macromolecular Material Factory | Wang Guidong Song Rui | 2015 | 2015 | pending | whole biomass |
| 7 | China | Algae protein/polylactic acid (PLA) biodegradable blending material and preparation method thereof | CN105038167A | Micro | Cynaobacteria (*Spirulina* sp.) | Zhu Nianqing Wang Qing Chen Haiyan | Wuxi Taihu Lake Blue Algae Resources Application Technology Research Institute Co Ltd | 2015 | 2015 | 2016 | Poly(lactic acid) (PLA) |
| 8 | China | Method for preparing polyolefin algae plastic through microwaves | CN105330931A | Micro | Cyanobacteria | Zhou Fuhai Pan Qi |  | 2015 | 2016 | 2018 | whole biomass /polyolefin |
| 9 | China | Thermoplastic prepared from algae | CN105331063A | Micro | Cyanobacteria (*Microcystis aeruginosa, Anabena, Spirulina*) | Zhou Fuhai Pan Qi |  | 2015 | 2016 | pending | PHA PLA, poly-hydroxy methane, PHB, polyolefine |
| 10 | China | Method for preparing plastic from algae | CN105331034A | Macro |  | Zhou Fuhai Pan Qi |  | 2015 | 2016 | pending | whole biomass |
| 11 | China | Blue alga based compound biological plastic with nano-calcium nutrient enriched and preparation method of biological plastic | CN105907066A | Micro | Cyanobacteria | Chen Kexia Chen Keliang Chao Jianping | Tongling Founder Plastics Technology Co Ltd | 2016 | 2016 | pending | Polymer polyurethane |
| 12 | China | Layered nano filler montmorillonite modified cyanobacteria-based compound bioplastic and preparation method thereof | CN105968761A | Micro | Cyanobacteria | Chen Kexia Chen Keliang Chao Jianping | Tongling Founder Plastics Technology Co Ltd | 2016 | 2016 | withdrawn | polydactyl acid PLA |
| 13 | China | A kind of organic nano bentonite modified cyanophyceae base Biocomposite material and preparation method thereof | CN106117999A | Micro | Cyanobacteria |  | Tongling Founder Plastics Technology Co Ltd | 2016 | 2016 | withdrawn | PLA |
| 14 | China | Preparation method of microalgae-based polymer composite thin film | CN105860111A | Micro | Cyanobacteria and Green Microalgae (*Spirulina* and *Chlorella*) | Shi Bo Liangliang Guo Yongjun Chen Qiutong | Guangdong University of Petrochemical Technology | 2016 | 2016 |  | polyalcohol PVA |
| 15 | China | The biological plastics composition for preparing the method for the algae powder of protein content reduction and being prepared with the powder | CN108350178A | Macro | Macroalgae (Ulva lactuca) |  | Ira Nova Co | 2016 | 2018 | pending | whole biomass |
| 16 | China | A kind of degradable blue-green algae base biological plastics and preparation method thereof | CN107057247A | Micro | Cyanobacteria | Jiangsu Academy of Agricultural Sciences | Zhou Qing Han Shiqun Chang Yajun Wang Tao | 2017 | 2017 | pending | whole biomass lipid, starch, carbohydrate, PHB |
| 17 | China | The EVA expanded materials and preparation method of a kind of biomass of blue-green algae containing thermoplasticity | CN106867175A | Micro | Cyanobacteria | Tang Ailan |  | 2017 | 2017 | pending | Polymer Ethylene vinyl acetate |
| 18 | China | A kind of green composite foam material and preparation method thereof | CN109679135A | Micro | Cyanobacteria | Wang Wei Zhang Baikai Jiang Shuai Hua Tianyu Wu Quan Qian Yin Jiangang | Wuxi City Environmental Science And Technology Co Ltd, Jiangnan University | 2018 | 2019 | 2020 | whole biomass |
| 19 | China | Green alga environment-friendly sole material and preparation method thereof | CN110591405A | Micro | Green Microalgae (Chlorella) |  | DONGGUAN FUMA SHOES MATERIAL Co Ltd | 2019 | 2019 | pending | whole biomass |
| 20 | England | A packaging item | WO2021171016A1 | Macro |  | Pierre-Yves PASLIER, Rodrigo Garcia Gonzalez |  | 2020 | 2021 | 2021 | Polysaccharide |
| 21 | France | Edible bioplastic from seaweed and the manufacturing technology thereof | WO2014108887A2 | Micro | Macroalgae (*Eucheuma cottonii*, *Caulerpa l.entilli-fera*, S*argassum polycystum*) | Noryawati S. Si. MULYONO |  | 2012 | 2014 |  | whole biomass |
| 22 | France | A biodegradable plastic like material obtained from a seaweed | WO2016113716A1 | Macro | Macroalgae | Sambhu BHADRAMohamed GOUSERaghavendra BARKIBabu Padmanabhan |  | 2015 | 2016 |  | whole biomass |
| 23 | Germany | Material based on macroalgae | DE102020103185A1 | Macro | Macroalgae (*Ulva* spp, *Agarophyton vermiculophyllum*) | Ramona BosseFrederike ReimoldLaurie Carol HofmannBela H. BuckJoachim HenjesDieter W. HoffmannIna Enders | HOCHSCHULE BREMERHAVEN Nordsee GmbH Alfred Wegener Institut fur Polar und Meeresforschung | 2020 | 2021 | pending | whole biomass |
| 24 | India | Novel biodegradable polymer composition useful for the preparation of biodegradable plastic and a process for the preparation of said composition | AU2007245266A1 | Micro | Cyanobacteria | Supreethi Sumanam | BNT Force Biodegradable Polymers Pvt Ltd | 2007 | 2007 | 2013 | whole biomass |
| 25 | Italy | Process for Producing Starch From Microalgae | WO2017130106A1 | Micro | Microalgae | Pagnanelli Francesca , Toro Luigi , Di Caprio Fabrizio , Altimari Pietro | Bio-P S R L | 2016 | 2017 |  | Carbohydrates |
| 26 | Japan | Method for producing sheet material using microalgae | JP2004162209A | Micro | Green Microalgae | Naotaka Fujitani, Hiromi Seki, Akira Morikawa, Yuji Yamaguchi, Hiroyuki Takenaka | Microalgae Corporation | 2002 | 2004 | 2009 | whole biomass |
| 27 | Japan | Production method for polyhydroxyalkanoate using only photosynthesis | JP6492011B2 | Micro | Cyanobacteria | Matsui Minami Shio Kurihara Nyokushin Rauhoon Chun Pin Sudish Kumar | RIKEN Institute of Physical and Chemical Research, Universiti Sains Malaysia (USM) | 2014 | 2015 | 2019 | PHA |
| 28 | Japan | Method for producing plastic starting material and related substance from cyanobacteria | WO2015115520A1 | Micro | Cyanobacteria (*Synechocystis* sp.) | Takashi Koyamauchi Yumi Hirai Saito Kazuki Saito Ayuko Iijima Kuwahara |  | 2014 | 2015 |  | PHA |
| 29 | Japan | Method for producing plastic raw materials and related substances in cyanobacteria | JP5946080B2 | Micro | Cyanobacteria | Takashi Koyamauchi Takashi Koyamauchi Yumi Hirai Yumi Hirai Kazuki Saito Kazuki Saito Keiji Numata Numata |  | 2014 | 2016 | 2016 | PHA |
| 30 | Netherlands | Algal thermoplastics, thermosets, paper, adsorbants and absorbants | US9758757B2 | Micro | Prototheca moriformis |  | Corbion Biotech Inc | 2011 | 2017 | 2017 | TAG |
| 31 | South Korea | Seaweed fiber-reinforced biocomposite and method for producing the same using high-temperature grinding | EP2079794A1 | Macro | Macroalgae | Seong-Ok HanHong-Soo KimYoon-Jong YooYeong-Bum SeoMin-Woo Lee | Korea Institute of Energy Research KIER | 2006 | 2009 | 2012 | whole biomass |
| 32 | South Korea | Production of Polyhydroxyalkanoates from the Saccharified Solution of Hydrodictyaceae Algal Biomass | KR101293639B1 | Micro | Green Microalgae (Hydrodictyaceae) | Kim Jin-seok Park Si Jae Hwang Hyun-jin Choi Jeong-seop Lee Seung-hwan Song Bong-geun |  | 2011 | 2013 | 2013 | PHA |
| 33 | South Korea | Red algae-polylactic acid and manufacturing method thereof | KR101237880B1 | Macro | Red macroalgae | Seok-gu Seo Young-beom |  | 2012 | 2012 | 2013 | PLA |
| 34 | South Korea | Methods for Producing Organic Acids From the Saccharified Solution of Hydrodictyaceae Algal Biomass | KR20130099475A | Micro | Hydrodictyaceae | Kim Jin Seog , Kim Jin Cheol , Kim Yeong Un , Choi Gyung Ja , Lee Seung Hwan , Kim Seul Ki , Nguyen Mai Cuong , Park Myung Soo | Korea Res Inst Chem Tech | 2012 | 2013 | 2013 | Carbohydrates |
| 35 | South Korea | Seaweed-based food packaging coating | KR102233086B1 | Macro | Red macroalgae | Steven A. Santos |  | 2013 | 2015 | 2021 | whole biomass |
| 36 | South Korea | Filament composition for 3 dimensional print comprising red algae fiber | KR20170009425A | Macro |  | Seo Young-beom Heo Yoon-young |  | 2015 | 2017 |  | whole biomass |
| 37 | South Korea | Method of manufacturing bio plastic and bio plastic manufactured thereby | KR102270614B1 | Macro | Macralgae |  | Yoo Ha-neul | 2019 | 2020 | 2021 | Polymer |
| 38 | South Korea | Environmental-friendly polymer composition and method of fabricating the same | KR102160900B1 | MM | Micro/macroalgae consortia | Kim Byung-yong |  | 2020 | 2020 | 2020 | Polymer |
| 39 | South Korea | Eco-friendly plastic bag using seaweed and vegetable raw material and manufacturing method therefor | WO2021101094A1 | Macro | Macroalgae | Cha Wan-young |  | 2020 | 2021 | pending | Polymer |
| 40 | South Korea | Composition for manufacturing biodegradable plastic seaweed farming nets containing seaweed fertilizer ingredients | KR102393864B1 | MM | Macro/micro algae | Cho Cheon-rae |  | 2021 | 2022 | 2022 | Polymer |
| 41 | USA | Algae-blended compositions for thermoplastic articles | EP2424937B1 | Micro | Cyanobacteria and Green Microalgae (*Nannochloropsis* and *Spirulina*) | Bo Shi, James H. Wang | Kimberly Clark Worldwide Inc , Kimberly Clark Corp | 2009 | 2012 | 2015 | Polymer |
| 42 | USA | Use of marine algae for producing polymers | US20120165490A1 | Micro | Isochrysis | Scott R. Lindell Christopher M. Reddy | Marine Biological Laboratory , Woods Hole Oceanographic Institute WHOI ,Western Washington University | 2011 | 2012 | 2018 | Alkenones |
| 43 | USA | Algal thermoplastics, thermosets, paper, adsorbants and absorbants | CN104114689A | Micro | Green Microalgae (*Chlorella* sp.) | A. Haring, A. Yeskelayinen, J. Chiuru, C. René, T. Lithier, K. Natinen, J. Pere, S. Sousa, J. Pischoke, A. McKee, J. J. Chernowhouse, A. R. Pavlowski | TerraVia Holdings Inc | 2011 | 2014 |  | TAG |
| 44 | USA | Methods Of Bioplastic Production | US20130344550A1 | Micro | Green Microalgae (*Scenedesmus obliquus*) | Miller Charles , Rahman Asif , Sims Ronald , Sathish Ashik , Anthony Renil | Utah State University | 2012 | 2013 | Abandoned | Polymer |
| 45 | USA | Polymer compositions comprising algae materials | US20140273169A1 | Micro | Microalgae | Frederic Scheer, Kelvin T. Okamoto, William E. Kelly | Cereplast Inc | 2013 | 2014 | 2015 | Polymer |
| 46 | USA | Algae-Blended Compositions Without Plasticizers | AU2016243338B2 | MM | macro/micro algae | Michael Lawrence Gross, Ryan Webster HUNT, Bo Shi, Mark Ashton Zeller | Kimberly Clark Worldwide Inc , Kimberly Clark Corp | 2015 | 2017 | 2019 | whole biomass |
| 47 | USA | Algae-blended thermoplastic compositions | US20200283600A1 | MM | macro/micro algae | Bo Shi, Michael Lawrence Gross, Ryan Webster Hunt, Mark Ashton Zeller | ALGIX LLC | 2015 | 2020 | pending | whole biomass |
| 48 | USA | Algae-derived flexible foam, and method of manufacturing the same | US20170066893A1 | Macro | Red and brown macroalgae |  | Bloom Health Holdings LLC | 2015 | 2020 | pending | whole biomass |
| 49 | USA | Process For Preparing An Algal Powder Containing A Reduced Content Of Proteins, And Bioplastic Composition Formulated From Such A Powder | US20180258231A1 | Macro | Macroalgae (Ulva Armoricana) | Lavoisier Philippe , Pierre Ronan , Benoit Maud | Eranova | 2016 | 2018 | 2020 | Carbohydrates |
| 50 | USA | Algae-Derived Antimicrobial Plastic Substrates, And A Method Of Manufacturing The Same | US20170183469A1 | Macro |  | Falken Robert , Hunt Ryan , Zeller Ashton | Bloom Holdings Llc | 2016 | 2018 |  | whole biomass |
| 51 | USA | Algae-based bioplastics and methods of making | US20200263125A1 | Micro | Green Microalgae (*Chlamydomonas*  and *Chlorella*) | Naohiro Kato | Louisiana State University and Agricultural and Mechanical College | 2017 | 2020 |  | TAG |
| 52 | USA | Method to produce a polysaccharide gel by increasing the pH of the polysaccharide | US10907223B2 | Micro | Cyanobacteria | Mona Mirsiaghi, Eric Sundstrom, Deepti Tanjore, Todd Pray, Rocco L. Mancinelli ,David T. Smernoff | University of California, HELIOBIOSYS Inc | 2018 | 2021 | 2021 | Polymer |
| 53 | USA | Algae thermoplastic composition and process of making | WO2020237232A1 | MM | macro/micro algae | Mark Ashton Zeller, Ryan Hunt | ALGIX LLC | 2019 | 2020 |  | whole biomass |
| 54 | USA | Edible cup and method of making the same | US20210204562A1 | Macro | Red macroalgae | Chelsea BRIGANTI, Leigh Ann Tucker, Monica Bhatia, Kevin Stanton | Loliware Inc | 2021 | 2021 |  | Polymer |
| 55 | USA | Biodegradable and industrially compostable injection molded microcellular flexible foams, and a method of manufacturing the same | US11565448B2 | MM | macro/micro algae | Robert Falken | O2 Partners LLC | 2019 | 2022 |  | PLA Polymer |
